# Antibiotic Isoflavonoids, Anthraquinones, and Pterocarpanoids from Pigeon Pea (*Cajanus cajan* L.) Seeds against Multidrug-Resistant *Staphylococcus aureus*

**DOI:** 10.3390/metabo12040279

**Published:** 2022-03-23

**Authors:** Lex Aliko P. Balida, Julia Theresa A. Regalado, Jade Joshua R. Teodosio, Kathryn Ann H. Dizon, Zhe Sun, Zhao Qi Zhan, Jenny Marie D. Blancaflor, Jan Vincent N. Sollesta, Zenith M. Villorente, Jonel P. Saludes, Doralyn S. Dalisay

**Affiliations:** 1 Center for Chemical Biology and Biotechnology (C2B2), University of San Agustin, Iloilo City 5000, Philippines; labalida@usa.edu.ph (L.A.P.B.); jtregalado@usa.edu.ph (J.T.A.R.); jjteodosio@usa.edu.ph (J.J.R.T.); kadizon@usa.edu.ph (K.A.H.D.); 2 Shimadzu Asia Pacific (SAP), Singapore Science Park I, Singapore 118264, Singapore; sunzhe@shimadzu.com.sg (Z.S.); zhaoqi@shimadzu.com.sg (Z.Q.Z.); 3 Department of Pharmacy, College of Health and Allied Medical Professions, University of San Agustin, Iloilo City 5000, Philippines; jmblancaflor@usa.edu.ph; 4 Maridan Industries, Inc., Jaro, Iloilo City 5000, Philippines; jvsollesta@maridan.com.ph (J.V.N.S.); zmvillorente@maridan.com.ph (Z.M.V.); 5 Center for Natural Drug Discovery and Development (CND3), University of San Agustin, Iloilo City 5000, Philippines; jsaludes@usa.edu.ph; 6 Department of Chemistry, College of Liberal Arts, Sciences, and Education, University of San Agustin, Iloilo City 5000, Philippines; 7 Balik Scientist Program, Department of Science and Technology Philippine Council for Health Research and Development (DOST-PCHRD), Bicutan, Taguig City 1631, Philippines; 8 Department of Biology, College of Liberal Arts, Sciences, and Education, University of San Agustin, Iloilo City 5000, Philippines

**Keywords:** *Cajanus cajan*, pigeon pea, antibiotics, multidrug resistant *Staphylococcus aureus*, mass spectrometry, metabolomics, flavonoids, anthraquinones, pterocarpanoids

## Abstract

*Cajanus cajan* L. (pigeon pea, locally known in the Philippines as kadios) seed is a functional food with health benefits that extend beyond their nutritional value. *C. cajan* seeds contain highly diverse secondary metabolites with enriched beneficial properties, such as antibacterial, anticancer, and antioxidant activities. However, the antibacterial activities of secondary metabolites from Philippine-grown *C. cajan*, against multidrug-resistant *Staphylococcus aureus* have not been thoroughly described. Here, we investigated the in vitro antibacterial properties of *C. cajan* seed against multidrug-resistant *S. aureus* ATCC BAA-44 (MDRSA) and three other *S. aureus* strains (*S. aureus* ATCC 25923, *S. aureus* ATCC 6538, and coagulase-negative *S. aureus*) and, subsequently, identified the antibiotic markers against *S. aureus* strains using mass spectrometry. Secondary metabolites from *C. cajan* seeds were extracted using acetone, methanol, or 95% ethanol. Antibacterial screening revealed antibiotic activity for the *C. cajan* acetone extract. Bioassay-guided purification of the *C. cajan* acetone extract afforded three semi-pure high-performance liquid chromatography (HPLC) fractions exhibiting 32–64 µg/mL minimum inhibitory concentration (MIC) against MDRSA. Chemical profiling of these fractions using liquid chromatography mass spectrometry (LCMS) identified six compounds that are antibacterial against MDRSA. High-resolution mass spectrometry (HRMS), MS/MS, and dereplication using Global Natural Products Social Molecular Networking (GNPS)™, and National Institute of Standards and Technology (NIST) Library identified the metabolites as rhein, formononetin, laccaic acid D, crotafuran E, ayamenin A, and biochanin A. These isoflavonoids, anthraquinones, and pterocarpanoids from *C. cajan* seeds are potential bioactive compounds against *S. aureus*, including the multidrug-resistant strains.

## 1. Introduction

Antibiotics are utilized to inhibit or eliminate microbes that cause infections in humans. Unfortunately, the emergence of antibacterial resistance renders commercially available antibiotics ineffective [1,2]. One of the factors causing antibacterial resistance includes mutations, or the development of resistant genes induced by the misuse of antibiotic drugs. The alarming increase in the rate of bacterial resistance has triggered a global health security emergency and public health threat as new opportunities for antibiotics discovery and development are scarce [3]. 

Almost all recognized antibacterial compounds, including last-resort drugs, are becoming ineffective to their target pathogen, causing high morbidity and mortality due to infection [4]. The absence of new effective antibiotics further limits the preventive measures for infection during critical medical procedures, such as childbirth, organ transplant, management of diabetes, and chemotherapy [5].

Alarmingly, several bacterial strains have developed resistance against clinically useful and effective drugs, such as *Escherichia coli*, *Klebsiella pneumoniae,* and Methicillin-resistant *Staphylococcus aureus* (MRSA). The World Health Organization classified these pathogens as the three crucial bacterial strains of focus [5]. For *E. coli* and *K. pneumoniae*, resistance against β-lactam antibiotics, such as third-generation cephalosporins is already evident, complicating the treatment of diseases caused by these pathogens, such as urinary tract infection (UTI) [5]. For methicillin-resistant *S. aureus* (MRSA), the infection has now developed from hospital acquisition to community-acquired infections, such as through the skin or wounds. Thus, the imminent threats of antibiotic resistance caused by MRSA have prompted us to search for potentially novel antibiotics from various sources, such as plants.

The inherent secondary metabolites in plants have been studied to provide defense against phytopathogens [6], which could then be used against pathogenic bacteria in humans [7]. Their production is driven by evolution against animal predation by increasing resistance or defense [8]. Some examples include phenols, tannins, flavonoids, terpenes, and alkaloids, which exhibit antibacterial properties [9], thus, providing alternative sources of antibacterial compounds to combat pathogenic infections. 

Recently, studies have indicated that plant-based foods and functional foods are not only a promising source of nutritional and health benefits [10,11] but could also provide secondary metabolites as natural antibiotics [12,13,14,15]. Functional foods are typically consumed for a normal diet [16] with added health benefits on top of their high nutritional value. In the Philippines, there are numerous claims regarding several functional foods with antibacterial properties. These include coconut (*Cocos nucifera*), malunggay (*Moringa oleifera*), papaya (*Carica papaya*), mungbeen (*Vigna radiata*), and kadios (pigeon pea, *C. cajan*) [17]. Despite numerous studies on these functional foods, further investigations on the active secondary metabolites and chemical properties to render such biological claims are lacking.

As part of our natural product drug discovery program [18,19,20,21], we investigated the seeds of a local plant, *C. cajan*, also known as “kadios” in the Philippines. The *C. cajan* plant is commonly grown as food, particularly for its seed, in selected locations in the Philippines as well as India, Indonesia, Myanmar, and Pakistan [22]. In Iloilo Province in the Philippines, *C. cajan* seed is a common ingredient in KBL (Kadios, Baboy, and Langka), a nutritional bean-based soup high in carbohydrate and protein [23]. Furthermore, there are limited studies regarding the antibacterial properties of *C. cajan* seeds. Although recently, secondary metabolites, such as an isoflavonoid cajanol [24] and a coumarin cajanuslactone [25] isolated from *C. cajan* roots and leaves, have shown antibacterial activities against *S. aureus* and *E. coli*. 

Additionally, reports regarding *C. cajan* seeds from the Philippines are generally about their nutritional [26,27] and agricultural values [28,29]. One study reported that Philippine *C. cajan* seeds contain chemical components, such as amino acids, polyphenols, trypsin inhibitors, lectins or phytohemagglutinins, flatulence factors, and phytates [30]. No reports have indicated the antibacterial activities of these metabolites. Hence, this study investigates the antibacterial properties of *C. cajan* seed extracts and identifies metabolites eliciting the activity against strains of *S. aureus*, including a multidrug-resistant strain.

## 2. Results

### 2.1. Preliminary Antibacterial Screening of C. cajan Extracts

To determine the solvent that is efficient to extract the antibacterial metabolites in *C. cajan*, we extracted the seeds with acetone, methanol, or 95% ethanol using the method described in Section 4.4. The acetone extract (20 mg/well) named KADA showed a zone of inhibition (ZOI) of 13.2 ± 1.7 mm against MDRSA. Subsequent testing of the acetone extract against other *S. aureus* strains, showed antibacterial activity against *S. aureus* ATCC 25923 and *S. aureus* ATCC 6538, with average ZOIs of 11.8 and 11.7 mm, respectively (Table 1). 

The antibacterial testing via agar well diffusion assay of the methanolic extract at 20 mg/well showed 13.3 ± 2.1 mm ZOI against MDRSA and 10.0 mm against *S. aureus* ATCC 25923. The 95% ethanol extract showed no activity at 20 mg/well and 40 mg/well but exhibited 12.8 mm ZOI at higher concentration of 90 mg/well. Due to its high inhibitory concentration against the multidrug-resistant strain, the ethanolic extract was not tested against the three other strains of *S. aureus*. Overall, the acetone extract revealed broader antibacterial properties against *S. aureus* strains compared to the methanolic and ethanolic extracts. Thus, we focused on acetone extract for subsequent bioassay guided purification. 

### 2.2. Bioassay Guided Purification of Acetone Crude Extract

**Figure 1:**
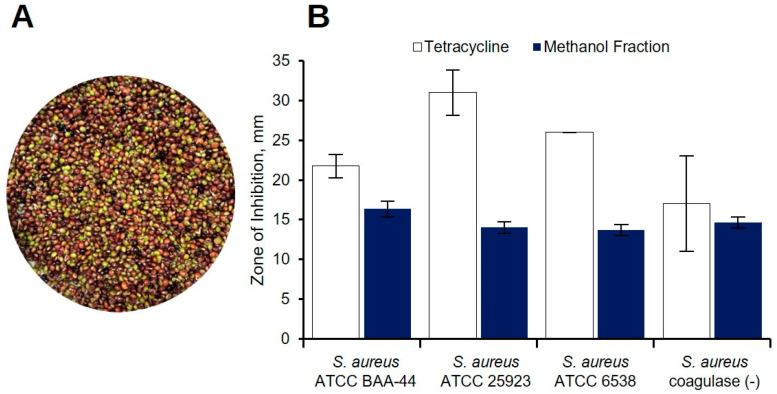
Antibacterial screening of *C. cajan* methanol fraction against four *S. aureus* strains. (**A**) Photo of *C. cajan* seeds. (**B**) Zone of inhibition (±SD) of KADAM fraction at 20 mg/well against the multidrug-resistant *S. aureus* ATCC BAA-44, *S. aureus* ATCC 25923, *S. aureus* ATCC 6538, and *S. aureus* coagulase (concentration of 20 mg/well, exhibiting), evaluated using agar well diffusion assay. Tetracycline was used as the positive control at a concentration of 0.25 mg/well against *S. aureus* ATCC BAA-44, *S. aureus* ATCC 25923, and *S. aureus* ATCC 6538 and 0.05 mg/well against *S. aureus* coagulase (concentration of 20 mg/well, exhibiting). DMSO was used as the negative control. Error bars represent the standard deviation. The assay was conducted in triplicates.

Purification of *C. cajan* seed acetone extract named KADA was guided by antibiotic assays, primarily against the multidrug-resistant *S. aureus* ATCC BAA-44 (MDRSA) (Figure S1). Partitioning of dried KADA between methanol and hexane separated the secondary metabolites from *C. cajan* seeds (Figure 1A) based on polarity. The KADAM fraction exhibited antibacterial activity against all *S. aureus* strains tested: (1) *S. aureus* ATCC BAA-44 (MDRSA), (2) *S. aureus* ATCC 25923, (3) *S. aureus* ATCC 6538, and (4) *S. aureus* coagulase (−) at a concentration of 20 mg/well, exhibiting ZOI between 11 to 16.5 mm (Figure 1B). No ZOI was evident in the KADAH fraction. (Figure S2A).

Moving to flash column chromatography as the next purification step, we attempted to reconstitute dried KADAM in polar and semi polar organic solvents, such as acetone, methanol, ethanol, dichloromethane, and ethyl acetate. However, the sample showed poor solubility with these solvents. Notably, KADAM completely dissolved in water, which led to another partitioning of the sample between water and ethyl acetate that resulted to ethyl acetate and aqueous fractions named KADAME and KADAMW, respectively. The antibacterial testing of both fractions against MDRSA at 20 mg/well via agar well diffusion assay showed 21.4 mm average ZOI in plates treated with fraction KADAME. The aqueous fraction KADAMW demonstrated no activity against MDRSA at 20 mg/well (Figure S2B).

**Figure 2:**
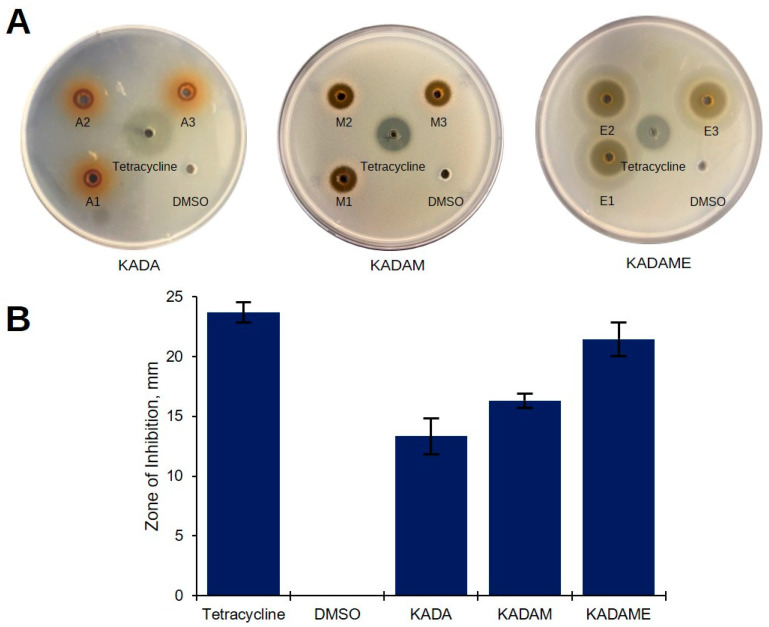
Antibacterial activity of solvent-partitioned extracts against the multidrug-resistant *S. aureus*. (**A**) Plates of agar well diffusion assay showing the clearing zone when treated with *C. cajan* seed acetone extract KADA (A1–A3), methanol fraction KADAM (M1–M3), and ethyl acetate fraction KADAME (E1–E3) at 20 mg/well against MDRSA. Tetracycline (0.25 mg/well) and DMSO were used as positive and negative controls, respectively. (**B**) Zones of inhibition of the samples in mm (±SD) against *S. aureus* ATCC BAA-44. Error bars represent the standard deviation.

Interestingly, the solvent partitioned extracts showed increasing antibacterial activity against MDRSA. The *C. cajan* seed extract KADA tested at 20 mg/well initially exhibited antibacterial properties against MDRSA, with an average of 13.2 mm ZOI (Figure 2A). Subsequently, solvent partitioning of KADA resulting to bioactive fraction KADAM and solvent partitioning of KADAM resulting to bioactive fraction KADAME showed clearing around the wells with 16.3 and 21.4 mm ZOIs, respectively (Figure 2A,B).

### 2.3. Purification of Ethyl Acetate Fraction (KADAME) by Flash Column Chromatography

**Figure 3:**
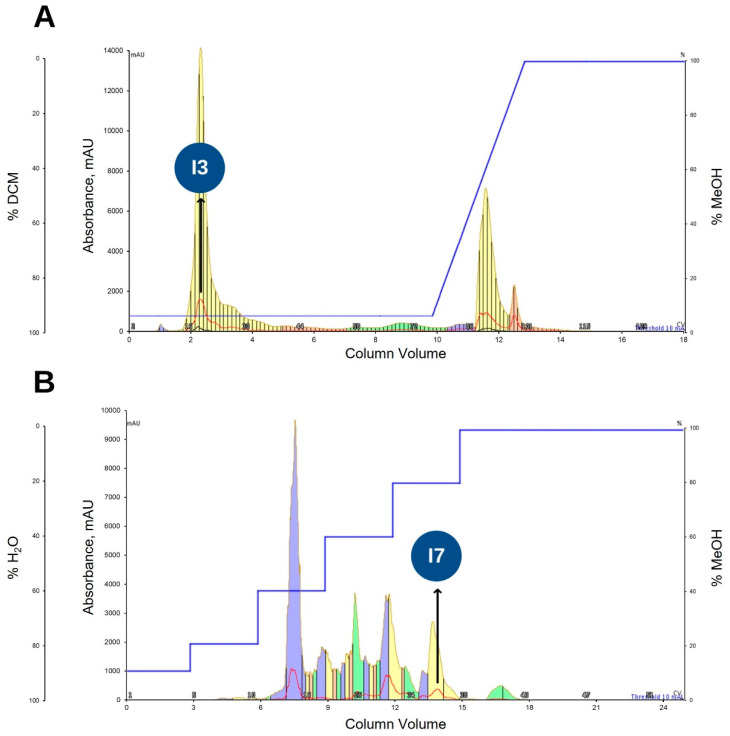
Bioassay-guided fractionation using flash column chromatography. (**A**) Chromatogram from the purification of KADAME fraction using normal phase chromatography. Peak annotated with black arrow as I3 in blue circle corresponds to the early eluting fraction KADAMEI3, which exhibited bioactivity against MDRSA. (**B**) Chromatogram from the subsequent purification of fraction KADAMEI3 using reversed phase flash column chromatography. Peak annotated with black arrow as I7 in blue circle corresponds to fraction KADAMEI3I7, which exhibited activity against MDRSA. UV λ_max_ 254 nm (red curve) and λ_max_ 365 nm (black curve).

The bioactive fraction KADAME obtained above was subjected to purification using normal phase flash column chromatography. The purification method described in Section 4.7 afforded 14 fractions. Early eluting fraction named KADAMEI3 (I3, annotated) (Figure 3A) was eluted at 95:5 DCM/MeOH and exhibited antibacterial activity against MDRSA as tested via TLC bioautography (Figure S3). Subsequently, the purification via the reversed phase flash column chromatography of fraction KADAMEI3 described in Section 4.8 afforded 8 fractions. Fraction KADAMEI3I7 (I7, annotated) (Figure 3B) was eluted at 2:8 H_2_O/MeOH and showed antibacterial activity against MDRSA as tested via TLC bioautography (Figure S4).

### 2.4. Antibiotic Kinetics Assay of KADAMEI3I7 Fraction

**Figure 4:**
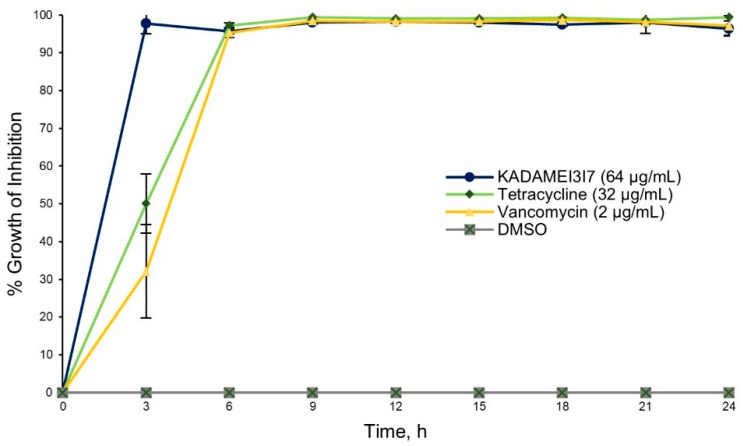
Antibiotic kinetics assay against MDRSA. The data illustrated are the % growth inhibition (± SD) of KADAMEI3I7 (64 µg/mL), tetracycline (32 µg/mL, positive control), vancomycin (2 µg/mL, positive control), and DMSO against *S. aureus* ATCC BAA-44 at different time interval. Bacterial density was measured every 3 h from 0 to 24 h. Assay was conducted in triplicates. Error bars represent the standard deviation.

The antibiotic kinetic profile of fraction KADAMEI3I7 (64 µg/mL) showed immediate antibacterial action against MDRSA as compared to tetracycline (32 µg/mL) and vancomycin (2 µg/mL) (Figure 4). Fraction KADAMEI3I7 also exhibited sustained antibacterial activity against MDRSA for 24 h. Complete growth inhibition (>90%) against MDRSA after 24 h, was observed at a minimum inhibitory concentration (MIC) of 64 µg/mL for fraction KADAMEI3I7, 32 µg/mL for tetracycline, and 2 µg/mL for vancomycin.

### 2.5. High Performance Liquid Chromatography (HPLC) Purification of KADAMEI3I7 and Antibacterial Assay against MDRSA

**Figure 5:**
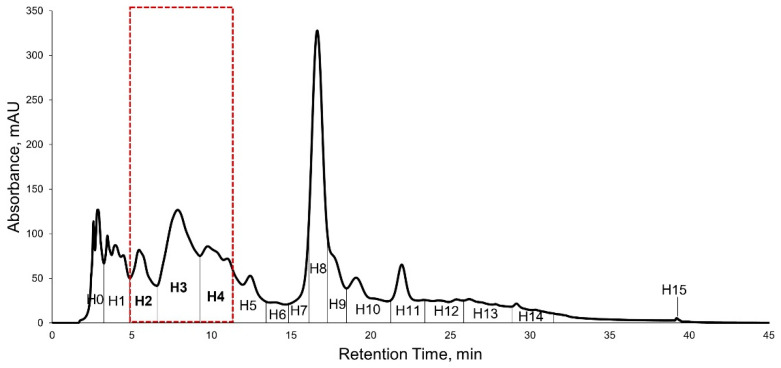
HPLC Chromatogram of KADAMEI3I7 (UV λ_max_ 254 nm, black curve line). HPLC Fractions H2, H3, and H4 (highlighted broken line) showed antibacterial activity against MDRSA.

KADAMEI3I7 was further purified using reversed-phase HPLC using the method described in Section 4.9. The HPLC purification separated the KADAMEI3I7 fraction into sixteen fractions, which were all detected at 254 nm, suggesting aromaticity (Figure 5).

**Figure 6:**
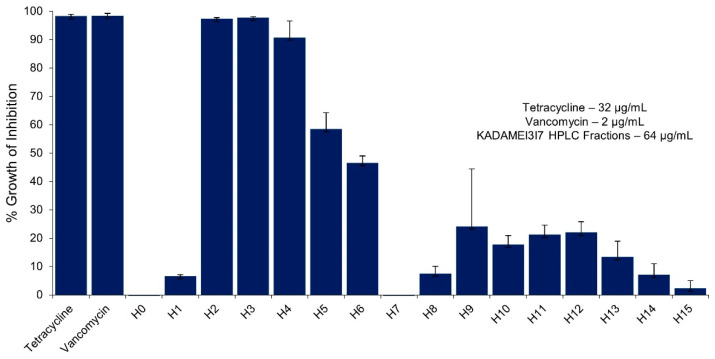
Microbroth susceptibility assay of sixteen KADAMEI3I7 HPLC fractions (64 µg/mL), tetracycline (32 µg/mL, positive control), and vancomycin (2 µg/mL, positive control) against MDRSA. The data illustrated are average % growth inhibition (± SEM) of the samples against *S. aureus* ATCC BAA-44. Error bars are standard error of the mean. Assay was conducted in three replicates and in three trials. KADAMEI3I7 is the mother fraction of the sixteen HPLC fractions tested.

The sixteen HPLC fractions obtained were tested at 64 µg/mL against MDRSA. The sample treated wells were compared with tetracycline (32 µg/mL) and vancomycin (2 µg/mL) as the positive control, and DMSO as the negative control. Three HPLC fractions exhibited complete growth inhibition (>90%) against MDRSA namely: (1) KADAMEI3I7H2, H3, and H4 (Figure 6), which corresponds to HPLC peaks highlighted in Figure 5. Fractions KADAMEI3I7H2, KADAMEI3I7H3, and KADAMEI3I7H4 exhibited complete growth inhibition after 24 h at minimum inhibitory concentration of 32 µg/mL for KADAMEI3I7H2 and 64 µg/mL for both KADAMEI3I7H3 and KADAMEI3I7H4.

### 2.6. Liquid Chromatography-Triple Quadrupole Mass Spectrometry Profiling, MS/MS Analysis, and Dereplication by Global Natural Products Molecular Networking Social (GNPS)

To gain insights into the identity of metabolites present in the three bioactive HPLC fractions, the chemical profile of fractions KADAMEI3I7H2, KADAMEI3I7H3, and KADAMEI3I7H4 was further investigated. The UV peaks from each of the bioactive fraction that eluted at the same retention time showed the same *m*/*z* values suggesting that these fractions contain six common metabolites (Figure S5), potentially eliciting the activity against MDRSA: Metabolite A (*m*/*z* 285.05 [M + H]^+^, RT 5.78 min), Metabolite B (*m*/*z* 269.10 [M + H]^+^, RT 5.86 min), Metabolite C (*m*/*z* 315.10 [M + H]^+^, RT 6.11 min), Metabolite D (*m*/*z* 355.15 [M + H]^+^, RT 6.4 min), Metabolite E (*m*/*z* 299.10 [M + H]^+^, RT 6.8 min), and Metabolite F (*m*/*z* 285.10 [M + H]^+^, RT 7 min). 

MS/MS analysis of the six *m/z* detected and dereplication using the libraries in Global Natural Products Social Molecular Networking (GNPS), identified Metabolite F (Figure S6) and Metabolite B as isoflavones biochanin A and formononetin (also known as biochanin B) (Figure S7), respectively. The experimental MS/MS spectra of Metabolites F and B shared at least 10 MS/MS fragments with their corresponding library hits, which obtained a cosine similarity score of 0.98 and 0.92, respectively. The MS/MS of four other *m*/*z* detected have no corresponding hit in GNPS, suggesting that these metabolites are potentially unknown or no similar compounds are present in the GNPS database, which warrants further analyses to confirm and establish their chemical identities.

### 2.7. High Resolution Mass Spectrometry (LCMS), MS/MS, and Dereplication Analysis 

The semi-pure HPLC fractions KADAMEI3I7H2 and KADAMEI3I7H3 were further investigated using High-Resolution Mass Spectrometry (HRMS) to support the above findings. The analysis accurately (<1.0 ppm) predicted the chemical formula of the measured *m*/*z* values of the six metabolites of interest in both fractions and provided their corresponding double bond equivalent (DBE) (Figures S8–S13), as presented in Table 2. Dereplication relying on ChemSpider database identified the six *m/z* values as anthraquinones: rhein (Metabolite A) and laccaic acid D (Metabolite C), isoflavones: isoformononetin (Metabolite B), biochanin A (Metabolite F), and ayamenin A (Metabolite E), and pterocarpanoid: crotafuran E (Metabolite D), in the HPLC fractions (Figures S14–S19). 

**Figure 7:**
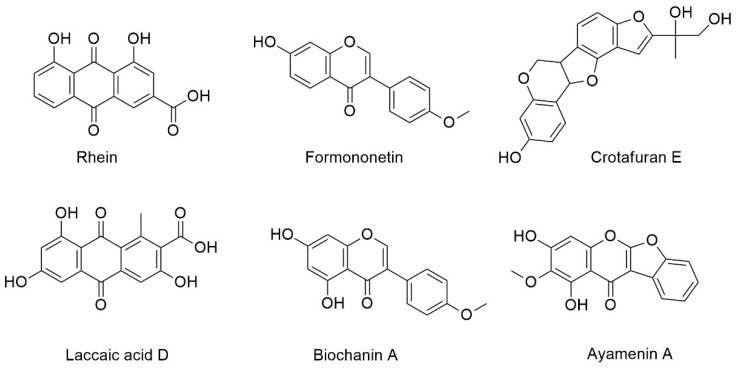
Putative identities and chemical structures of the six antibiotic metabolites of interest from *C. cajan* seeds.

Alternatively, dereplication using the NIST Library identified Metabolites B and F as formononetin and biochanin A (Figures S20 and S21), which corroborated our preliminary analysis using libraries in GNPS. The chemical IDs and structure of these compounds are presented in Figure 7.

To resolve the ambiguity of the mass spectrometry results for Metabolite B, we secured standards of the two isomers formononetin and isoformononetin and compared their MS/MS fragmentation using the method described in Section 4.10. MS/MS analysis with collision energy and concentration of standards and KADAMEI3I7H2 injected held constant, confirmed a similarity in fragmentation pattern between Metabolite B and formononetin, as isoformononetin showed distinct MS/MS spectra (Figure S22). Thus, the experiment unambiguously resolved Metabolite B as formononetin.

## 3. Discussion

Breakthroughs in natural products research have shifted the focus of drug-development efforts to mine antibacterial compounds derived from medicinal plants, such as *C. cajan* [31,32,33], as they display enriched bioactivities [34,35,36,37,38,39,40,41,42,43]. Plants as functional food offer metabolites with minimal side effects, making them advantageous for development as antibacterial agents [44,45]. 

Secondary metabolites produced by these plants arise from evolutionary processes to improve their protection against animal predation and pathogen invasion. Their production is derived from the interconnected metabolic pathways in plants [46], such as the phenylpropanoid [47], isoprenoid, and alkaloid metabolisms [48], which constitute a rich repertoire of chemical diversity that could be exploited for various applications, such as antibacterial agents for therapeutic purposes.

However, classical approaches in identifying small antibacterial molecules from complex matrices, such as plant extracts, have been proven a challenge. The small yield of active compounds limits the characterization of natural products from plants, which requires a large extraction throughput and laborious purification steps. Fortunately, the advent of state-of-the art technologies and novel techniques, such as mass spectrometry metabolomics, has expedited the dereplication or identification of these natural products even in minute amounts [49]. Metabolomics takes advantage of the presence of numerous chemical libraries and open-source databases, including proprietary platforms, developed to aid researchers in identifying these metabolites, such as NIST, ChemSpider, and GNPS, in the absence of spectroscopic techniques—the golden standard in natural product discovery.

In this study, we demonstrated that the Philippine grown *C. cajan* seeds showed antibacterial activity against *S. aureus* strains, including multidrug-resistant strain and coagulase-negative strain. The multidrug-resistant *S. aureus* ATCC BAA-44 was selected as the main test strain as this pathogen has grown resistant to 18 clinically effective antibiotics namely ampicillin, amoxicillin/clavulanic acid, ciprofloxacin, cephalothin, doxycycline, gentamicin, erythromycin, imipenem, methicillin, penicillin, tetracycline, oxacillin azithromycin, clindamycin, ceftriaxone, rifampin, amikacin, streptomycin, and tobramycin [50]. 

We also included a coagulase-negative strain *S. aureus* as one of the target organisms in the assay. Variants of coagulase-negative *S. aureus* have been isolated in many hospitals and are known to be virulent as those of *S. aureus* producing coagulase. The variants can colonize and spread infection among hospital patients [51,52,53]. Moreover, methicillin-resistant coagulase-negative *S. aureus* strains have been isolated from livestock industries [54,55,56] and pose risks in transmitting infection to humans.

Previously, a novel antibacterial isoflavone named cajanol was isolated from *C. cajan* roots, which exhibited antibacterial activity against *S. aureus* and *E. coli* by cell membrane damage and DNA cleavage [24]. Another novel coumarin cajanuslactone isolated from *C. cajan* leaves also exhibited antibacterial activity against *S. aureus* at 0.031 mg/mL minimum inhibitory concentration (MIC) [31]. However, these two antibiotic metabolites were not detected in this study. 

Instead, six metabolites were identified using metabolomics from the semi-pure HPLC fractions that elicited the activity against MDRSA. Two metabolites, i.e., formononetin, also known as biochanin B, and biochanin A belonging to the class isoflavones, were unambiguously identified by MS, MS/MS, and dereplication analyses in comparison with the standard compounds. The putative identities of four metabolites revealed that these are known plant metabolites—namely, rhein and laccaic acid D belonging to the class anthraquinones, crotafuran E belonging to the class pterocarpanoids, and isoflavonoid ayamenin A as determined by MS, MS/MS, and dereplication analyses. 

Confirmation of their identities requires nuclear magnetic resonance analysis or comparison with the standard compounds. Nonetheless, mass spectrometry and dereplication analyses enabled us to identify the antibiotic biomarkers in *C. cajan* seeds. The isoflavones biochanin A and biochanin B were known to be present in *C. cajan* leaves [57,58] and are not unique in *C. cajan* seeds. Biochanin A and biochanin B were also isolated from *Trifolium baccarinii* Chiov. [59] and red propolis [60], suggesting that these compounds are widely distributed among the Fabaceae family, including *C. cajan*. 

Although plants in the Fabaceae family also produce anthraquinones [61] and pterocarpanoids [62], no reports on the isolation of rhein, laccaic acid D, crotafuran E, and ayamenin A from *C. cajan* were declared. In north-east India and southern Rajasthan, various tropical plants, such as *Schleichera oleosa* (Sapindaceae), *Butea monosperma* (Fabaceae) [63] and *C. cajan* (Fabaceae) are suitable breeding grounds for lac insect *Kerria lacca* (Kerr), which produce laccaic acid, also known as lac dye [64].

Interestingly, three of these compounds particularly, biochanin A, biochanin B, and rhein, were reported to exhibit antibacterial properties against a wide array of pathogens. In numerous in vitro studies, biochanin A, including formononetin, has been demonstrated to possess antibacterial properties [65,66] against *E*. *coli, P. aeruginosa, S. enteritidis, K. pneumoniae,* and *S. aureus* [67]. However, antibacterial treatment with biochanin A alone resulted in only moderate activity against these pathogens [67,68,69], which led older studies to focus mainly on synergistic approaches, utilizing this isoflavone for combination treatment to restore ineffective antibiotics, such as ciprofloxacin and ofloxacin [70,71]. 

In contrast, a more recent study showed promising antibacterial activity of biochanin A against MRSA clinical isolates, *P. aeruginosa,* and *E. coli* whereby biochanin A exhibited 32–128 µg/mL MIC against these pathogens [59]. Moreover, it was demonstrated previously that biochanin A exhibited 64 µg/mL minimum inhibitory concentration (MIC) against *S. aureus* ATCC 25923 [70], which corroborates the findings in our study.

Attempts to elucidate the mechanism of action of biochanin A against selected pathogens were also reported. In MRSA, biochanin A inhibited the production of α-hemolysin, which affects the capacity of bacteria to infect hosts, in a dose-dependent manner [72]. A recent report showed that biochanin A improved defense against *Salmonella* infection both in vivo and in vitro by activating AMPK/ULK1/mTOR-mediated autophagy and macrophage extracellular traps (METs) [73]. It was also found to inhibit DNA synthesis and flagella formation in *Xanthomonas axonopodis* pv. *glycines* (Xag), a known plant pathogen, and altered bacterial membrane composition [74].

On the other hand, emerging studies on pharmacological effects of rhein suggested that it could provide alternative antisepsis [75], and antibacterial treatments to diseases caused by pathogenic infections [76,77,78]. Rhein was discovered to have promising antibacterial properties against various strains of MRSA, which exhibited a minimum inhibitory concentration of 62.5–250.0 µg/mL. 

It suppressed the expression of *mecA/mecI/mecR1* and *blaZ/blaI/blaR1* genes in SCCmec, causing a decreased resistance of MRSA to β-lactam antibiotics [79]. In in vitro experiments, rhein showed good antibacterial activity against 21 *S. aureus* strains in which a total of 88 genes were identified to be differentially regulated by this compound [80]. Combination therapies using rhein with ampicillin and oxacillin was also found to exhibit synergistic or partially synergistic activity against MRSA [81].

The biological activities of laccaic acid D, ayamenin A and crotafuran E, have not been extensively studied, and no reports exist regarding their antibacterial properties. Laccaic acid D possesses intermediate antioxidant activity at 0.1, 0.2 and 0.5 mg/mL [82] as tested by DPPH assay. Its methylated derivative also has antifungal activity against some phytopathogenic fungi, such as *Alternaria solani, Curvularia lunata, Erysiphe pisi, Helminthosporium oryzae,* and *Verticillium* sp., which commonly cause foliar diseases in crop plants [83]. 

On the other hand, ayamenin A was recently isolated from *Iris songarica* belonging to the Iridiceae family, which exhibited estrogenic activity [84]. Whereas crotafuran E and its analogs isolated from *Crotalaria pallida*, a close relative of *C. cajan* in the Fabaceae family exhibited antioxidant and anti-inflammatory activities [62,85].

In summary, our bioassay guided fractionation of the *C. cajan* acetone extract isolated three HPLC fractions, which exhibited 32–64 µg/mL minimum inhibitory concentration against the multidrug-resistant *S. aureus*. Mass spectrometry, MS/MS, and dereplication using the ChemSpider database, NIST, and libraries in GNPS identified six metabolites common in these bioactive fractions. 

These molecules are the isoflavones biochanin A, biochanin B, and ayamenin A; anthraquinones rhein and laccaic acid D; and pterocarpanoids crotafuran E. Our findings demonstrated that functional foods, such as pigeon pea, are a rich resource for mining antibiotic metabolites against pathogenic *S. aureus,* including the multidrug-resistant strains.

## 4. Materials and Methods

### 4.1. Plant Material

Drought-resistant and short-duration type *C. cajan* L. Millsp. seeds of the Monahan variety, was planted in Barangay Anabo, Lemery, Iloilo, Philippines in a 5000 m^2^ lot. The seeds were directly seeded in a raised bed soil with a depth of 1 in. at 1 × 1 m apart. To promote a healthy plant growth, the soil was enhanced with Nitrogen-Phosphorus-Potassium (NPK) fertilizer. Irrigation was performed twice weekly. The pods were handpicked on the fifth month when a pod color change from green to brown was observed. Then, the harvested pods were manually dehulled to recover the seeds. The collected seeds were authenticated by the Philippine National Museum Botany Division and transported to the University of San Agustin, Gen. Luna St., Iloilo City, Philippines.

### 4.2. Reagents and Standards

Standards formononetin and isoformononetin were secured from Changzhou Guiding Bio-Tech. Co., Ltd. (Changzhou, China). Tetracycline (Sigma-Aldrich Co.^®^, St. Louis, MO, USA) and vancomycin hydrochloride (United States Pharmacopeia Reference Standard) were used as a positive control for antibacterial assays. Acetone, methanol, ethanol, and hexane used for extraction and solvent partitioning were obtained from Sigma-Aldrich Co.^®^ (St. Louis, MO, USA), while ethyl acetate was purchased from RCI Labscan^®^ Limited (Bangkok, Thailand). Dichloromethane used in the flash column purification was purchased from Thermo Fisher Scientific^®^ Co. (Waltham, MA, USA). Water and methanol (HPLC-grade) from RCI Labscan^®^ Limited (Bangkok, Thailand) were used as mobile phase for HPLC purification. Water, acetonitrile, and formic acid (MS-grade) were purchased from Sigma-Aldrich Co. ^®^ (St. Louis, MO, USA).

### 4.3. Test Pathogens

Four *Staphylococcus* strains: (1) *S. aureus* ATCC BAA-44 (MDRSA), (2) *S. aureus* ATCC 25923, (3) *S. aureus* ATCC 6538, and (4) *S. aureus.* coagulase (−) were used to test the antibacterial activity of *C. cajan* seed extracts. These test organisms were grown and maintained at 37 °C in tryptic soy agar for no more than 24 h until they were used for antibacterial assay.

### 4.4. Phytochemical Extraction

Fresh batch of *C. cajan* seeds was thoroughly washed with tap water five times and distilled water three times to remove soil, stem, leaves, and other unwanted materials. The washed seeds were air-dried at room temperature for 4 h, and then ground for 20 s using an industrial grinder to increase the surface area for an efficient maceration. Then, the powdered seeds were macerated using acetone, methanol, or 95% ethanol. A total of 47 kg *C. cajan* seeds was extracted with acetone. 

Extraction was performed thrice to maximize the recovery of bioactive secondary metabolites. In the first extraction, 3 L of solvent was added per 1 kg of seeds, with a 1:1 plant-material-to-solvent ratio for the succeeding two extractions. The ground seeds were macerated in the solvents for 18–24 h prior to collection of extracts. The collected extracts were filtered twice, concentrated in vacuo using the rotary evaporator (35 °C and 180 rpm), then dried using the centrifugal evaporator and freeze dryer. The extract was then subjected for antibacterial screening through agar well diffusion assay.

### 4.5. Solvent Partitioning of C. cajan Seed Extracts

Dried *C. cajan* acetone extract KADA was dissolved in methanol and partitioned with equal volume of hexane. Addition of hexane and partitioning was repeated until a clear hexane layer was obtained. The resulting methanol and hexane fractions were named KADAM and KADAH, respectively. Then, the fractions were concentrated in vacuo using the rotary evaporator (35 °C and 180 rpm) and dried using the centrifugal evaporator to afford 654.79 g (1.39% yield) of the bioactive fraction KADAM and 35.26 g (0.08% yield) of non-active fraction KADAH.

### 4.6. Solvent Partitioning of C. cajan Methanol Fraction

Due to poor solubility of the resulting dried methanol fraction KADAM in organic solvents (acetone, methanol, 95% ethanol, dichloromethane, and ethyl acetate), another solvent partitioning with water and ethyl acetate was performed. The dried KADAM fraction was reconstituted in water to form a completely homogeneous solution. Then, the solution was added with ethyl acetate at 1:2 (solution/ethyl acetate), swirled, and allowed to partition for 3 min. 

Partitioning was repeated until a clear ethyl acetate layer was obtained. The resulting ethyl acetate and aqueous fraction were then named KADAME, and KADAMW, respectively. Ethyl acetate fraction was concentrated in vacuo using the rotary evaporator (35 °C and 180 rpm) and dried using the centrifugal evaporator to afford 10.61 g (0.02% yield) KADAME. The aqueous fraction KADAMW was placed in a −80 °C freezer overnight and lyophilized in the freeze dryer. The fractions were then tested against *S. aureus* ATCC BAA-44, which revealed activity in fraction KADAME.

### 4.7. Purification of Ethyl Acetate Fraction by Accelerated Chromatographic Isolation (ACI™) Technology Biotage^®^ Isolera Normal Phase Flash Column Chromatography

A 1.0 g *C. cajan* ethyl acetate fraction KADAME was reconstituted in EtOAc and loaded directly on the Biotage^®^ SNAP Ultra 10 g normal phase samplet. The samplet was dried in a vacuum concentrator at 30 °C, then packed in a Biotage^®^ SNAP Ultra 50 g cartridge (Biotage^®^ HP-Sphere™ 25 µm, Biotage, Uppsala, Sweden). A flash chromatography method was developed based on the TLC profile of the KADAME fraction in different ratios of DCM and MeOH. 

The mobile phase was pumped at 40 mL/min using the following elution scheme: (1) isocratic elution with 95:5 DCM/MeOH at 10 column volumes (CV), (2) linear gradient elution from 95:5 DCM/MeOH to MeOH (3CV), and (3) flushing with MeOH (6 CV). Fractions were collected based on the peaks generated at λ_max_ 254 nm. A total of 14 fractions were obtained and subjected to TLC bioautography against *S. aureus* ATCC BAA-44. Pooled fractions were then concentrated in the rotary evaporator (35 °C and 180 rpm) and dried in the centrifugal evaporator. A total of 1.0019 g (0.002% yield) of the bioactive fraction named as KADAMEI3 was obtained.

### 4.8. Purification of Fraction KADAMEI3 by Accelerated Chromatographic Isolation (ACI™) Technology Biotage^®^ Isolera Reversed Phase Flash Column Chromatography

A 100 mg of the fraction KADAMEI3 obtained above was reconstituted in methanol and loaded directly on a SNAP Ultra 1.2 g C18 samplet. The samplet was dried in a vacuum concentrator at 30 °C and then packed in a Biotage^®^ SNAP Ultra 12 g C18 cartridge (Biotage^®^ HP-Sphere C18 25 µm, Biotage, Uppsala, Sweden). A flash chromatography method was developed based on the TLC profile of KADAMEI3 in 100% methanol. 

A constant flow rate at 12 mL/min of the mobile phase was used (H_2_O/MeOH), and a step gradient elution was conducted starting from: (1) 9:1 H_2_O/MeOH at 3 Column Volume (CV), (2) 8:2 H_2_O/MeOH at 3 CV, (3) 6:4 H_2_O/MeOH at 3 CV, (4) 4:6 H_2_O/MeOH at 3 CV, (5) 2:8 H_2_O/MeOH at 3 CV, and (6) Methanol at 10 CV. Fractions were collected in 12 mL test tubes and pooled based on the peaks generated at λ_max_ 254 nm. A total of eight fractions were obtained and subjected to TLC bioautography against *S. aureus* ATCC BAA-44. Pooled fractions were then concentrated in the rotary evaporator at 35 °C and 180 rpm, and dried using the centrifugal evaporator. A total of 83.8 mg (0.0002% yield) of the bioactive fraction named KADAMEI3I7 was obtained.

### 4.9. High-Performance Liquid Chromatography (HPLC) Purification of Bioactive Fraction

The bioactive fraction KADAMEI3I7 at 70.8 mg was reconstituted in 1 mL HPLC-grade methanol. Then, syringe filtration was performed to remove undissolved samples using a Teflon disk syringe filter (0.2 µm). One hundred µL of the filtered solution was injected into the HPLC unit (Shimadzu LC-20AD, Shimadzu, Kyoto, Japan) equipped with a degasser, an autosampler, column oven, variable wavelength detector, and a fraction collector. In total, ten sample injections were performed. A phenyl hexyl column (Phenomenex Luna^®^ 10 μm PREP Phenyl Hexyl 10, 250 × 10 mm) preceded by a guard column was used as the stationary phase. 

The column was held in the oven at 30 °C. The HPLC method was developed based on the retention of KADAMEI3I7, which was eluted at 2:8 H_2_O/MeOH from the step-gradient elution in the reversed phase flash column chromatography. The mobile phase was therefore, pumped at 5 mL/min starting with isocratic elution of 70% MeOH for 15 min then followed by 70–100% MeOH for 13 min, 100% MeOH for 7 min, 70–100% MeOH for 1 min, and back to 70% MeOH for 9 min. The fraction collector was simulated to pool fractions based on the retention of peaks generated at λ_max_ 254 nm. Pooled fractions were then dried using the centrifugal evaporator. The total run time covered 45 min and afforded 16 HPLC fractions.

### 4.10. Liquid Chromatography-Triple Quadrupole Mass Spectrometry (LC-TqMS) Profiling and Dereplication by Global Natural Products Social Molecular Networking (GNPS)

The chemical profile of the bioactive HPLC fractions named KADAMEI3I7H2, KADAMEI3I7H3, and KADAMEI3I7H4, was investigated by Liquid Chromatography-Triple Quadrupole Mass Spectrometry (LC-TqMS). A 100 µL solution of the sample (<100 µg) in 7:3 MeOH/H_2_O was prepared in a 0.2 mL micro-insert contained in an LC-MS clear screw vial. Two µL of each fraction was then injected into the LC-system (Shimadzu LCMS-8045, Kyoto, Japan) where the oven temperature was set at 40 °C, and UV detection of the eluted compounds was set and monitored at λ_max_ 254 nm. 

The separation of compounds based on their polarity was conducted using the Phenomenex Synergi™ (4 µm Hydro 80 Å, 2 × 100 mm) C18 column as the stationary phase through a gradient elution with water (Solvent A) and acetonitrile (MeCN) (Solvent B), each containing 0.1% formic acid (FA). The mobile phase at 20% MeCN was pumped for 1 min at a constant flow rate of 0.3 mL/min to the column to start the elution. Then, the MeCN concentration was ramped up linearly to 100% for 13 min. Flushing was set at 3 min, and reconditioning of the column by gradually decreasing the % MeCN to 20% was performed at 2 min, which was followed by a re-equilibration with 20% MeCN for 1 min. 

The compounds eluted at different retention times were then subjected for mass scanning by a mass spectrometer equipped with an Electrospray Ionization (ESI) and a triple-quadrupole mass analyzer. Third quadrupole (Q3) scans of the positive and negative ions covering a mass ion range of *m*/*z* 200–800 were then conducted to generate the mass spectra of the chemical constituents in the sample.

The MS parameters were set as follows: the nebulizing gas flow was set at 2.0 L/min, while the heating and drying gas flows were set at 10 L/min. The desolvation line temperature, interface temperature, and heat block temperature were set at 250, 300, and 400 °C, respectively. The event time was set at 0.20 s, and the total run time covered 20 min.

MS/MS analyses for selected mass ions in the positive mode detected in fractions KADAMEI3I7H2 and KADAMEI3I7H3 were then conducted at varying Collision Induced Dissociation (CID) Energy. Acquired MS/MS data files (.lcd) were converted to .mzML using MSConvert [86] to transform spectra from profile to centroid mode. The .mzML files were then uploaded to the Global Natural Products Social Molecular Networking (GNPS) through the Library Match Tool where, the precursor ion tolerance was set at 1 Da, MS/MS fragment ions at 0.5 Da with at least six minimum fragments shared and a cosine score >0.70 [87]. Two separate dereplication jobs are available from the Reference list [88,89].

### 4.11. Liquid Chromatography-Quadrupole Time-of-Flight (LC-QToF) Mass Spectrometry and MS/MS

Chemical profiling of fractions KADAMEI3I7H2 and KADAMEI3I7H3 by High-Resolution Mass Spectrometry (HRMS) was performed using Shimadzu LCMS-9030 Quadrupole-Time-of-Flight (Q-TOF) mass spectrometer equipped with dual-pump delivery system (LC-30AD), degasser (DGU-20A5R), auto-injector (SIL-30AC), and column oven (CTO-20AC). Five µg of sample was reconstituted in 500 µL 7:3 MeOH/H_2_O. Twenty µL of the analyte was injected into the LC system. 

The separation of compounds based on their polarity was conducted using the Phenomenex Synergi™ 2.5 µm Hydro-RP 100 Å (3.0 × 100 mm) as the stationary phase through a gradient elution with water (Solvent A) and methanol (MeOH) (Solvent B), each containing 0.1% formic acid (FA). The column was held in an oven at 40 °C. The mobile phase at 20% MeOH was pumped for 1 min at a constant flow rate of 0.65 mL/min to start the elution. Then, the MeOH concentration was ramped up linearly to 100% for 13 min. 

Flushing was set at 3 min, and reconditioning of the column by gradually decreasing the % MeOH to 20% was conducted at 2 min, which was followed by a re-equilibration step with 20% MeOH for 1 min. The compounds eluted at different retention times were ionized using the Heated Electrospray Ionization (HESI). The MS parameters were set as follows: the nebulizing gas flow was set at 2.0 L/min, while the heating and drying gas flow were set at 10 L/min. The desolvation line temperature, interface temperature, and heat block temperature were set at 250, 300, and 400 °C, respectively.

Mass scans in the positive mode covering a mass ion range of *m*/*z* 100–500 was performed to generate the mass spectra of the chemical constituents in the sample. Six precursor ions were subjected for MS/MS analysis at Collision Energy (CE) of −30 to −40 V spread 17 V. The dwell time and loop time were set at 0.02 s/event and 0.37 s/data point, respectively. The total acquisition covered 20 min. Chemical ID of MS/MS fragments were dereplicated using LabSolutions Insight Explore^®^ software, which rely on ChemSpider database and fragment peak annotation (Assign^®^). Dereplication using The National Institute of Standards and Technology (NIST) Library was also conducted.

### 4.12. Antibacterial Testing 

#### 4.12.1. Agar Well Diffusion Assay

The antibacterial activity against the multidrug-resistant *S. aureus* ATCC BAA-44, *S. aureus* ATCC 25923, *S. aureus* ATCC 6538, and *S. aureus* coagulase (−) of the *C. cajan* seed extracts was evaluated by agar-well diffusion assay. Overnight grown bacterial culture was inoculated in 5 mL Mueller–Hinton Broth (MHB) and adjusted to an optical density of 1 × 10^6^ CFU/mL as measured using a microplate reader (ELx808IU/BioTek Instruments, Inc., Winooski, VT, USA). The bacterial suspensions were then mixed with 1% Mueller–Hinton Agar (MHA) and dispensed into sterile Petri dishes. The mixture was allowed to solidify, and wells were made using a sterile borer. The seed extract (20 mg/well), DMSO (negative control) and tetracycline 5 mg/mL (positive control) were dispensed into the wells. The plates were then incubated at 37 °C for 18–24 h. The zones of inhibition were measured in millimeters (mm) using a caliper.

#### 4.12.2. Thin Layer Chromatography (TLC) Bioautography

TLC bioautography was conducted to qualitatively determine and investigate which of the fractions from the flash chromatography purification have an activity against *S. aureus* ATCC BAA-44. A 1 mL aliquot of each of the pooled fractions was dried using the centrifugal evaporator and then reconstituted in 10 µL of methanol for antibacterial screening against *S. aureus* ATCC BAA-44. Each of the 10 µL solution was loaded at 1.4 cm × 2.3 cm apart on a 7 cm × 7 cm normal phase silica TLC plate using a micropipette. 

The loaded plate was placed in a Petri dish and overlaid with 0.8% Mueller–Hinton Agar (MHA) containing bacterial cells (ca. 1 × 10^6^ CFU/mL) from overnight grown bacterial cultures. The plate was then incubated at 37 °C for 18–24 h. Three mL of resazurin (1.5 mg/mL) was flooded on the TLC plate to visualize the zone of inhibition. A blue coloration indicates antibacterial activity.

#### 4.12.3. Minimum Inhibitory Concentration (MIC) Assay

The guidelines and interpretation of the Clinical and Laboratory Standards Institute (CLSI) were followed for the minimum inhibitory concentration (MIC) determination. Isolated colonies of the multidrug-resistant *S. aureus* ATCC BAA-44 were transferred to Mueller–Hinton Broth (MHB), and cultures were grown then adjusted to a cell density of approximately 10^6^ CFU/mL. A 10-fold dilution of the sample was conducted in a 96-well plate, which was subsequently inoculated with MHB containing the pathogen to achieve a starting concentration equal to 512 µg/mL. The inoculated 96-well plate was then incubated at 37 °C. The optical density was measured at 600 nm using a microplate reader after 24 h. The % growth of inhibition was then calculated using Equation 1,

(1) $$\%\;\mathrm{growth}\;\mathrm{of}\;\mathrm{inhibition}=\left(\frac{\left(Negative\;Control-Sample\right)}{Negative\;Control}\right)*100,$$

The lowest sample concentration that exhibited a complete growth inhibition (>90%) was taken as the MIC of the sample.

#### 4.12.4. Antibiotic Kinetics Assay

Following the same procedure as the minimum inhibitory concentration assay, the antibiotic kinetics of the bioactive fraction KADAMEI3I7 was tested against the *S. aureus* ATCC BAA-44. Tetracycline and vancomycin were used as positive controls, while DMSO was used as the negative control. Bacterial densities were measured every 3 h (0, 3, 6, 9, 12, 15, 18, 21, 24) within a period of 24 h using a microplate reader at 600 nm.

#### 4.12.5. Microbroth Susceptibility Assay

Sixteen dried HPLC fractions were tested against the multidrug-resistant *S. aureus* ATCC BAA-44 based on the minimum inhibitory concentration of fraction KADAMEI3I7 (64 µg/mL). A 2.56 mg/mL stock concentration of each fraction was prepared by adding 78 µL of DMSO to 200 µg samples. Five µL of the stock solution was dispensed in a 96-well plate in triplicates. Then, 195 µL of bacterial cell suspension was added to each plate to achieve 64 µg/mL. 

For comparison, tetracycline (32 µg/mL) and vancomycin (2 µg/mL) were used as positive controls, while DMSO was used as the negative control. Mueller–Hinton Broth (MHB) was dispensed in the wells and used as the blank. The plate was then incubated for 18–24 h at 37 °C. The optical density was measured at 600 nm using a microplate reader, and the % growth inhibition was then calculated using Equation 1. The samples with greater than 90% growth inhibition were identified as the bioactive HPLC fractions.

## 5. Conclusions

Our study demonstrated that extracts from Philippine-grown pigeon pea seeds (*C. cajan* L.) were antibacterial against *S. aureus*, including the multidrug-resistant strain, which provides treatment possibilities for diseases caused by these pathogens, such as persistent skin infections. The presence of antibacterial activity against all four test *S. aureus* strains suggests that the isoflavones biochanin A, biochanin B, and ayamenin A; anthraquinones rhein and laccaic acid D; and pterocarpanoids crotafuran E from pigeon pea seeds are potential antibiotics against pathogenic *S. aureus* bacteria. 

Furthermore, the untapped potential of pigeon pea seeds as a rich resource of antibacterial metabolites is a potential reinforcement for our antimicrobial arsenal amidst the depleting antibiotic pipeline, providing alternative sources, i.e., functional foods for mining metabolites of medical importance. Thus, the exploration of other functional foods for bioactive metabolites is vital in drug discovery and development.

## Figures and Tables

**Table 1:** Antibacterial screening of *C. cajan* seed extracts against *S. aureus* strains.

Solvent/ Sample	Zone of Inhibition, mm			
	*S. aureus* ATCC BAA-44	*S. aureus* ATCC 25923	*S. aureus* ATCC 6538	*S. aureus* Coagulase (−)
Acetone ^a^	13.2 ± 1.7	11.8 ± 1.1	11.7 ± 0.5	–
Methanol ^a^	13.3 ± 2.1	10.0	–	–
95% Ethanol ^b^	12.8 ± 0.3	Not Tested	Not Tested	Not Tested
Tetracycline	23.5 ± 2.7 ^c^	29.0 ± 2.5 ^c^	26.8 ± 1.0 ^c^	14.0 ^d^
DMSO	–	–	–	–

Samples or control tested at ^a^ 20 mg/well, ^b^ 90 mg/well, ^c^ 0.25 mg/well, and ^d^ 0.05 mg/well, (–) No activity.

**Table 2:** HRMS profile of the six *C. cajan* seed metabolites showing the retention time, exact masses, chemical formulae, double bond equivalent, and putative identities.

Compound	Retention Time (min)	Measured Mass *m*/*z*	Ion	Formula Predictor	*Δppm*	*DBE* *	Chemical ID by MS/MS
A	5.78	285.0392	[M + H]^+^	C_15_H_8_O_6_	−0.47	12	Rhein ^a^
B	5.86	269.0809	[M + H]^+^	C_16_H_12_O_4_	0.16	11	Formononetin ^b,c^
			[M + H]^+^		0.16	11	Isoformononetin ^a^
C	6.11	315.0499	[M + H]^+^	C_16_H_10_O_7_	0.06	12	Laccaic acid D ^a^
D	6.4	355.1178	[M + H]^+^	C_20_H_18_O_6_	0.49	12	Crotafuran E ^a^
E	6.8	299.0549	[M + H]^+^	C_16_H_10_O_6_	-0.23	12	Ayamenin A ^a^
F	7	285.0757	[M + H]^+^	C_16_H_12_O_5_	-0.16	11	Biochanin A ^a,b,c^

* Double Bond Equivalent (DBE), ^a^ ChemSpider and Fragment Peak annotation (Assign^®^), ^b^ National Institute of Standards and Technology (NIST), ^c^ Global Natural Products Social Molecular Networking (GNPS).

## Data Availability

The data presented in this study are available in the article and supplementary materials.

## Supplementary Materials

The following supporting information can be downloaded at: https://www.mdpi.com/article/10.3390/metabo12040279/s1. Figure S1: Isolation tree showing the bioassay-guided purification of *C. cajan* acetone extract. Figure S2: Antibacterial testing against the multidrug-resistant *S. aureus* (MDRSA). Figure S3. Qualitative antibacterial activity evaluation of fractions obtained from the purification of the ethyl acetate fraction KADAME. Figure S4: Qualitative antibacterial activity evaluation of fractions obtained from the purification of fraction KADAMEI3. Figure S5: LC-MS Profile of the bioactive HPLC fractions. Figure S6: Dereplication of Metabolite F using LC-TqMS data and Global Natural Products Social Molecular Networking (GNPS). Figure S7: Dereplication of Metabolite B using LC-TqMS data and Global Natural Products Social Molecular Networking (GNPS). Figure S8: Chemical formula prediction of Metabolite A. Figure S9: Chemical formula prediction of Metabolite B. Figure S10: Chemical formula prediction of Metabolite C. Figure S11: Chemical formula prediction of Metabolite D. Figure S12: Chemical formula prediction of Metabolite E. Figure S13: Chemical formula prediction of Metabolite F. Figure S14: Dereplication of Metabolite A using High-Resolution Mass Spectrometry (HRMS) data and ChemSpider database. Figure S15: Dereplication of Metabolite B using High-Resolution Mass Spectrometry (HRMS) data and ChemSpider database. Figure S16: Dereplication of Metabolite C using High-Resolution Mass Spectrometry (HRMS) data and ChemSpider database. Figure S17: Dereplication of Metabolite D using High-Resolution Mass Spectrometry (HRMS) data and ChemSpider database. Figure S18: Dereplication of Metabolite E using High-Resolution Mass Spectrometry (HRMS) data and ChemSpider database. Figure S19: Dereplication of Metabolite F using High-Resolution Mass Spectrometry (HRMS) data and ChemSpider database. Figure S20: Dereplication of Metabolite F using High-Resolution Mass Spectrometry (HRMS) data and the National Institute of Standards and Technology (NIST) Library. Figure S21: Dereplication of Metabolite B using High-Resolution Mass Spectrometry (HRMS) data and the National Institute of Standards and Technology (NIST) Library. Figure S22: Comparison of MS/MS Fragmentation Pattern of Metabolite B from fraction KADAMEI3I7H2 with standards of isomers formononetin and isoformononetin.
