# Supplementary material for: Antibiotic Isoflavonoids, Anthraquinones, and Pterocarpanoids from Pigeon Pea (Cajanus cajan L.) Seeds against Multidrug-Resistant Staphylococcus aureus
Source: Metabolites. 2022 Mar 23;12(4):279. doi: 10.3390/metabo12040279 (PMC9030341; doi:10.3390/metabo12040279)
Supplement: Supplementary file 1 [file metabolites-12-00279-s001.zip › Supplementary.pdf]

Supporting Information

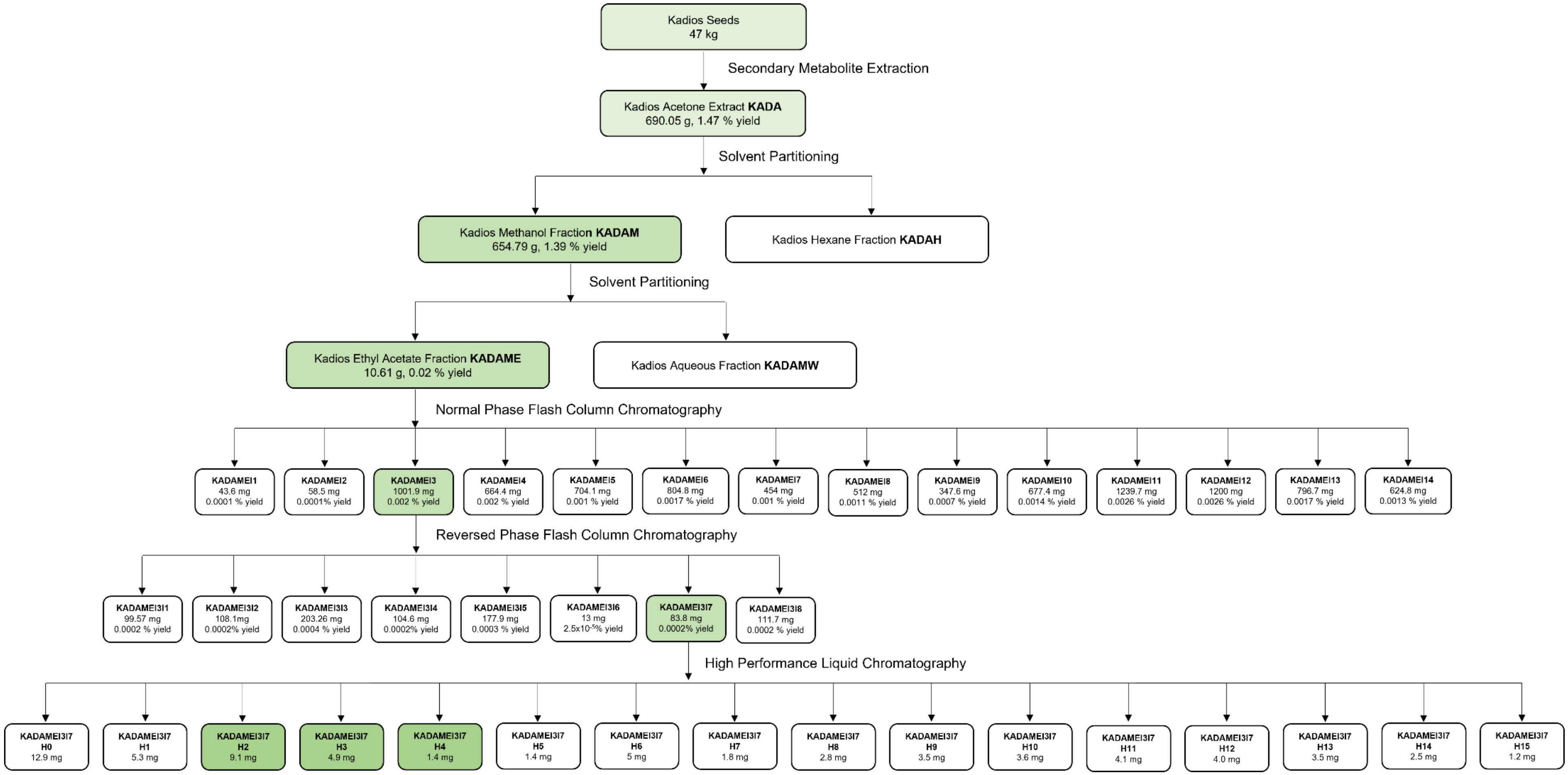

Figure S1: Isolation tree showing the bioassay-guided purification of *C. cajan* acetone extract. Boxes in shades of green are bioactive against *S. aureus* ATCC BAA-44. “Kadios” is the Filipino term for *C. cajan* seeds.

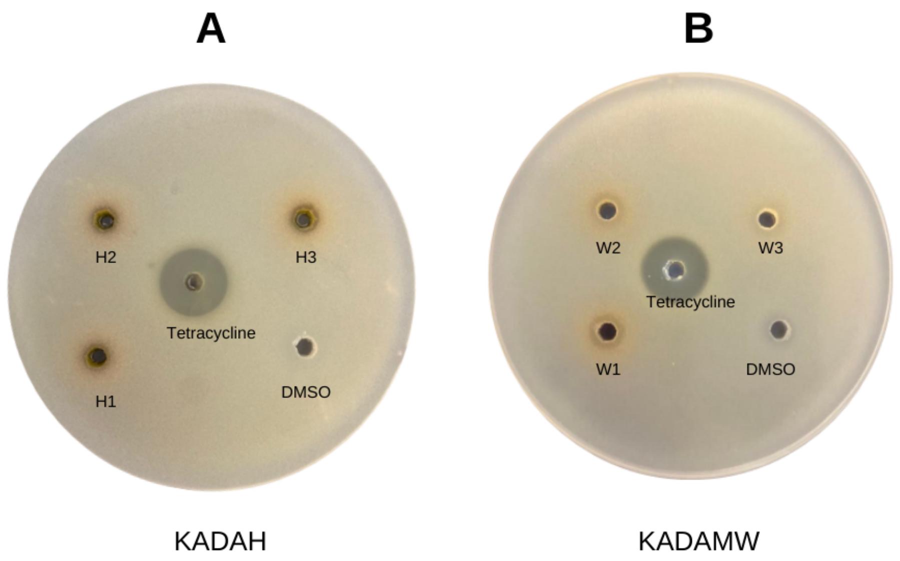

Figure S2: Antibacterial testing against the multidrug-resistant *S. aureus* (MDRSA). (**A**) Agar well diffusion assay of *C. cajan* hexane fraction KADAH (H1-H3) and *C. cajan* aqueous fraction KADAMW (W1-W3); (**B**)at 20 mg/well showing no zone of inhibition against *S. aureus* ATCC BAA-44. Tetracycline (0.25 mg/well) was used as positive control while DMSO was used as negative control.

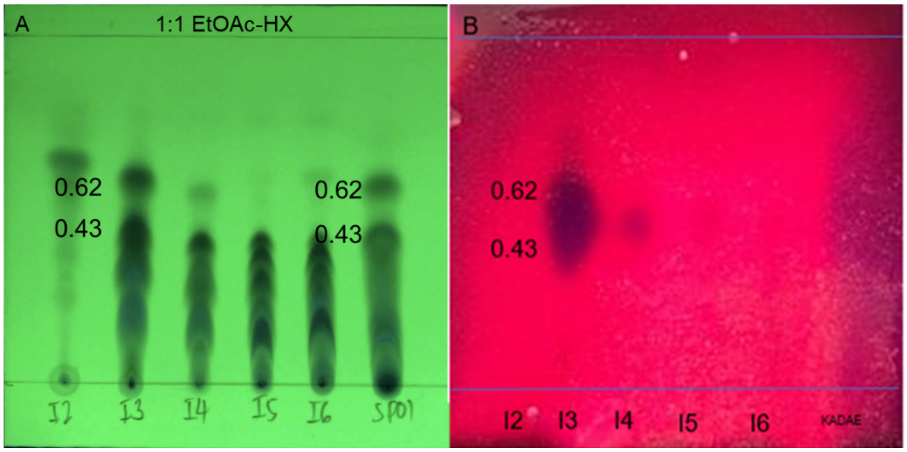

Figure S3. Qualitative antibacterial activity evaluation of fractions obtained from the purification of the ethyl acetate fraction KADAME. (**A**) Thin Layer Chromatography profiling of fractions KADAMEI2 to KADAMEI6 (I2-I6), obtained from the purification of ethyl acetate fraction, using normal phase silica TLC plate, resolved in 1:1 ethyl acetate-hexane, and visualized under UV 254 nm; (**B**) TLC Bioautography against *S. aureus* ATCC BAA-44 ($1x10^6$ CFU/mL), Resazurin (1.5 mg/mL), (Blue) Dead Cells, (Pink) Viable Cells. Antibacterial activity was observed in fraction KADAMEI3 labeled as I3.

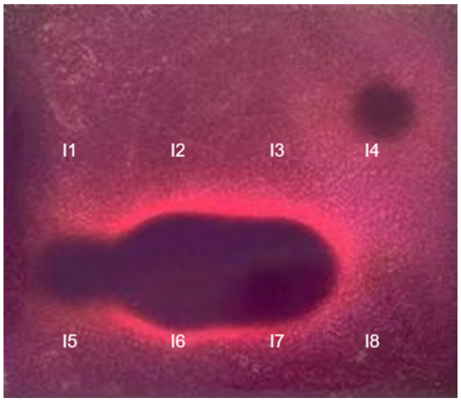

Figure S4. Qualitative antibacterial activity evaluation of fractions obtained from the purification of fraction KADAMEI3. TLC Bioautography of resulting 8 fractions (I1-I8) from the purification of fraction KADAMEI3 against *S. aureus* ATCC BAA-44 ($1x10^6$ CFU/mL), Resazurin (1.5 mg/mL), (Blue) Dead Cells, (Pink) Viable Cells. Antibacterial activity was observed in fraction KADAMEI3I7 labeled as I7.

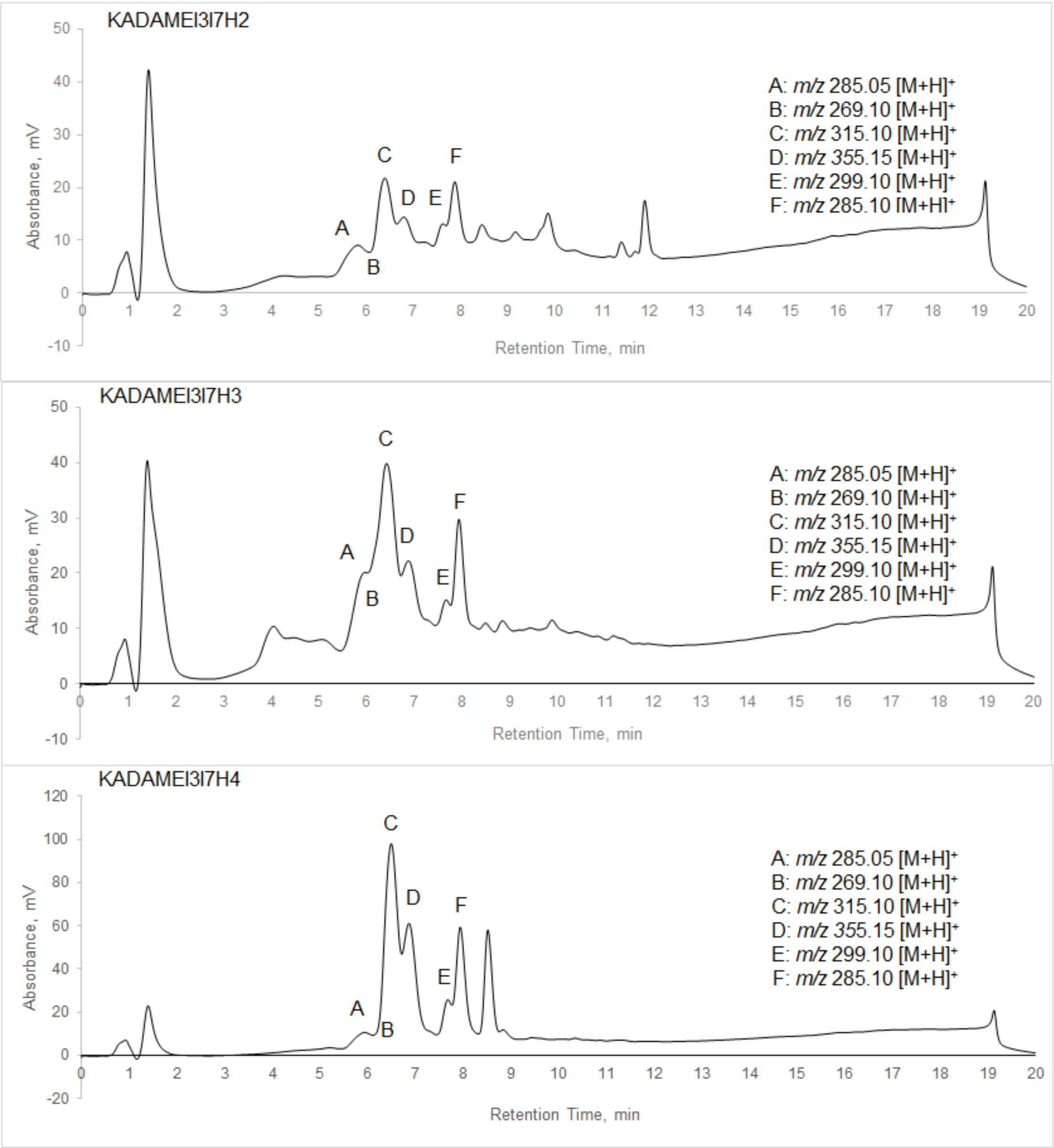

Figure S5. LC-MS Profile of the bioactive HPLC fractions. Stacked UV chromatogram at λ_{max} 254 nm of KADAMEI3I7H2, KADAMEI3I7H3, and KADAMEI3I7H4 acquired from Shimadzu LC-TqMS showing the overlapping of peaks and the similarity of compounds detected under each peak. Phenomenex Synergi™ 4 µm Hydro 80 Å (100 X 2 mm). (Stationary Phase), H_2O/Acetonitrile + 0.1 % Formic Acid (Mobile Phase)

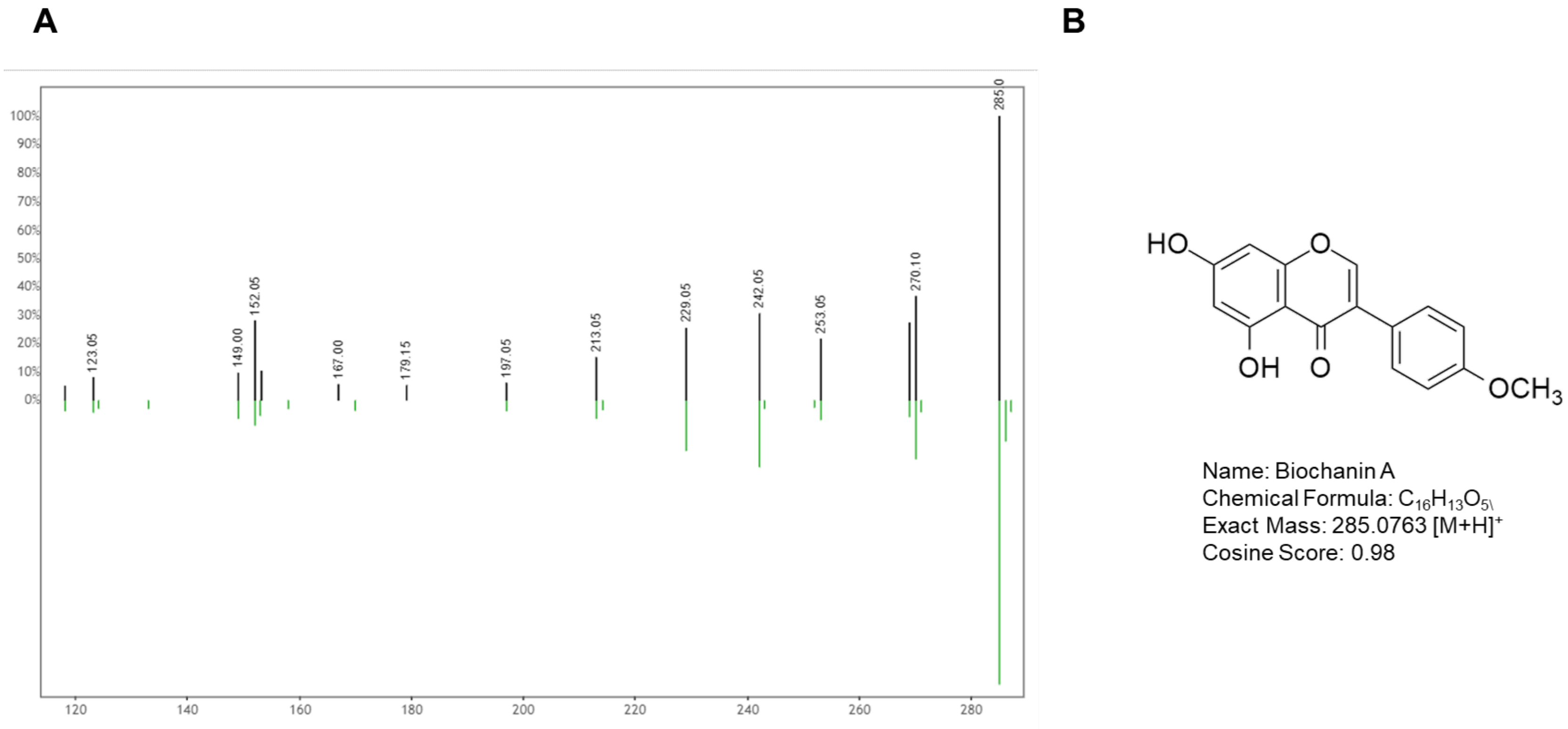

Figure S6. Dereplication of Metabolite F using LC-TqMS data and Global Natural Products Social Molecular Networking (GNPS). (**A**) The mirror match feature of GNPS showing the similarity of fragment peaks of the experimental (top, black) and library data (bottom, green).; (**B**) Identity of Metabolite F dereplicated as biochanin A with cosine similarity score of 0.98.

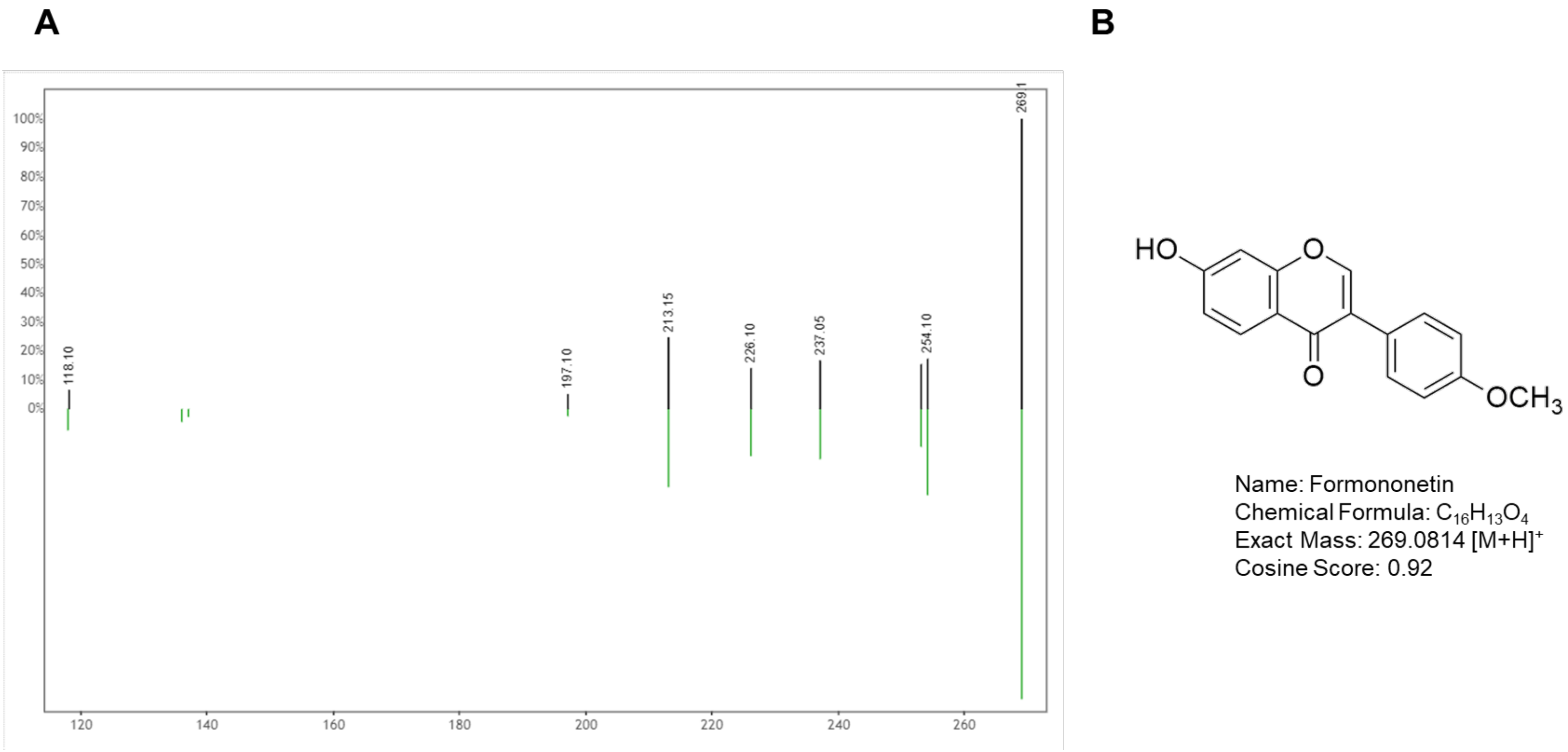

Figure S7. Dereplication of Metabolite B using LC-TqMS data and Global Natural Products Social Molecular Networking (GNPS). (**A**) The mirror match feature of GNPS showing the similarity of fragment peaks of the experimental (top, black) and library data (bottom, green).; (**B**) Identity of Metabolite B dereplicated as formononetin with cosine score of 0.92.

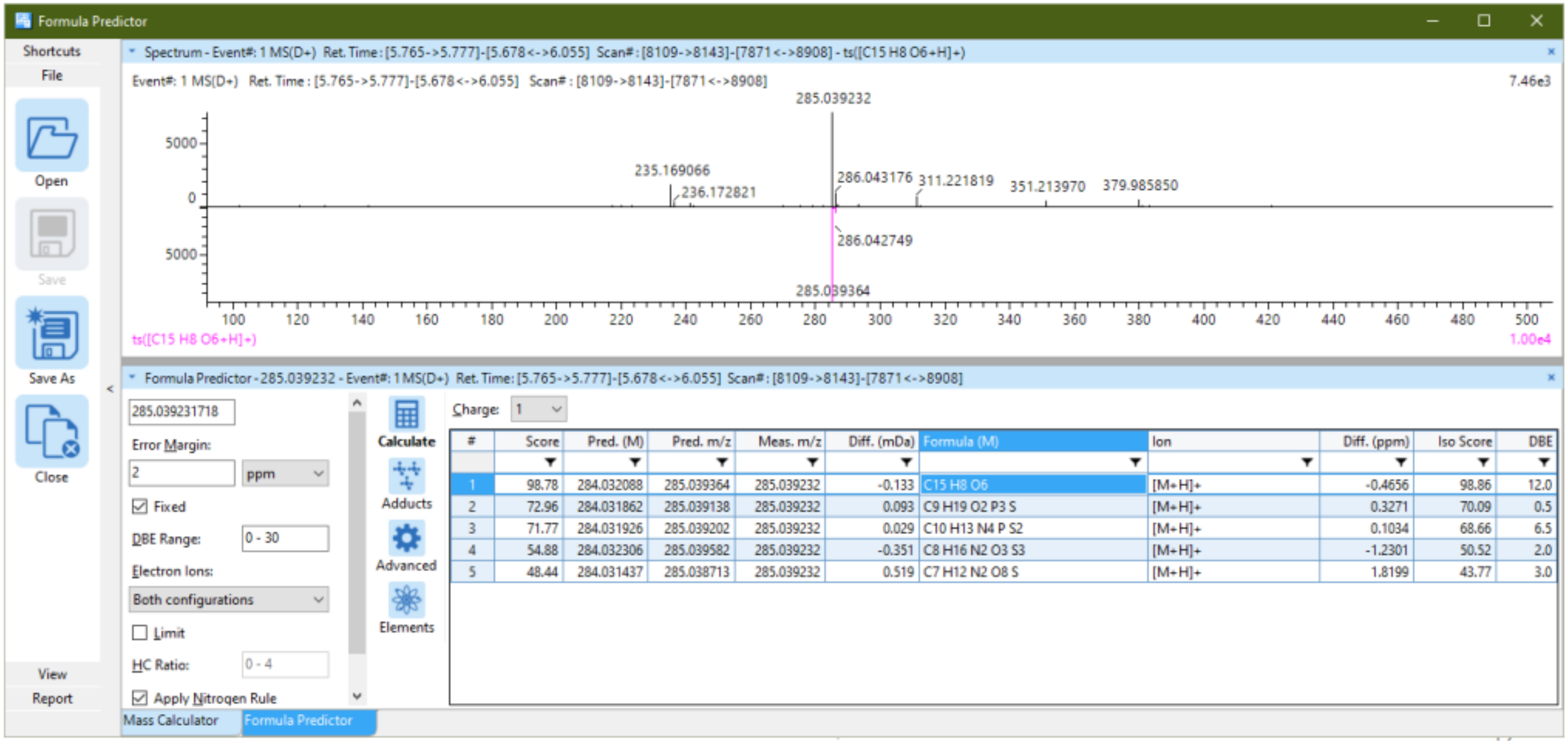

Figure S8. Chemical formula prediction of **Metabolite A**. Chemical formula was accurately predicted (< 1.0 ppm) based on the measured mass of **Metabolite A** (*m/z* 285.0392 [M+H]+).

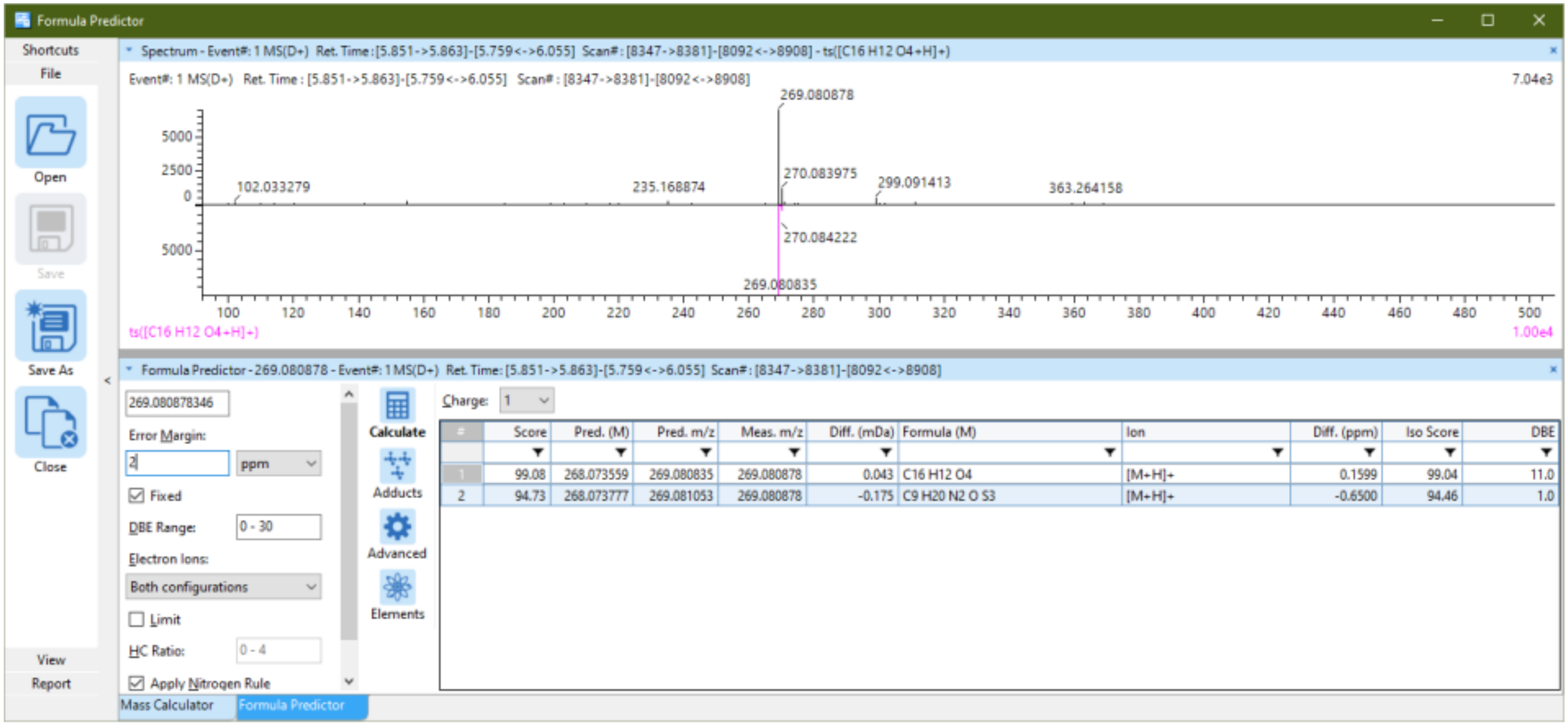

Figure S9. Chemical formula prediction of **Metabolite B**. Chemical formula was accurately predicted (< 1.0 ppm) based on the measured mass of **Metabolite B** (*m/z* 269.0809 [M+H]$^+$).

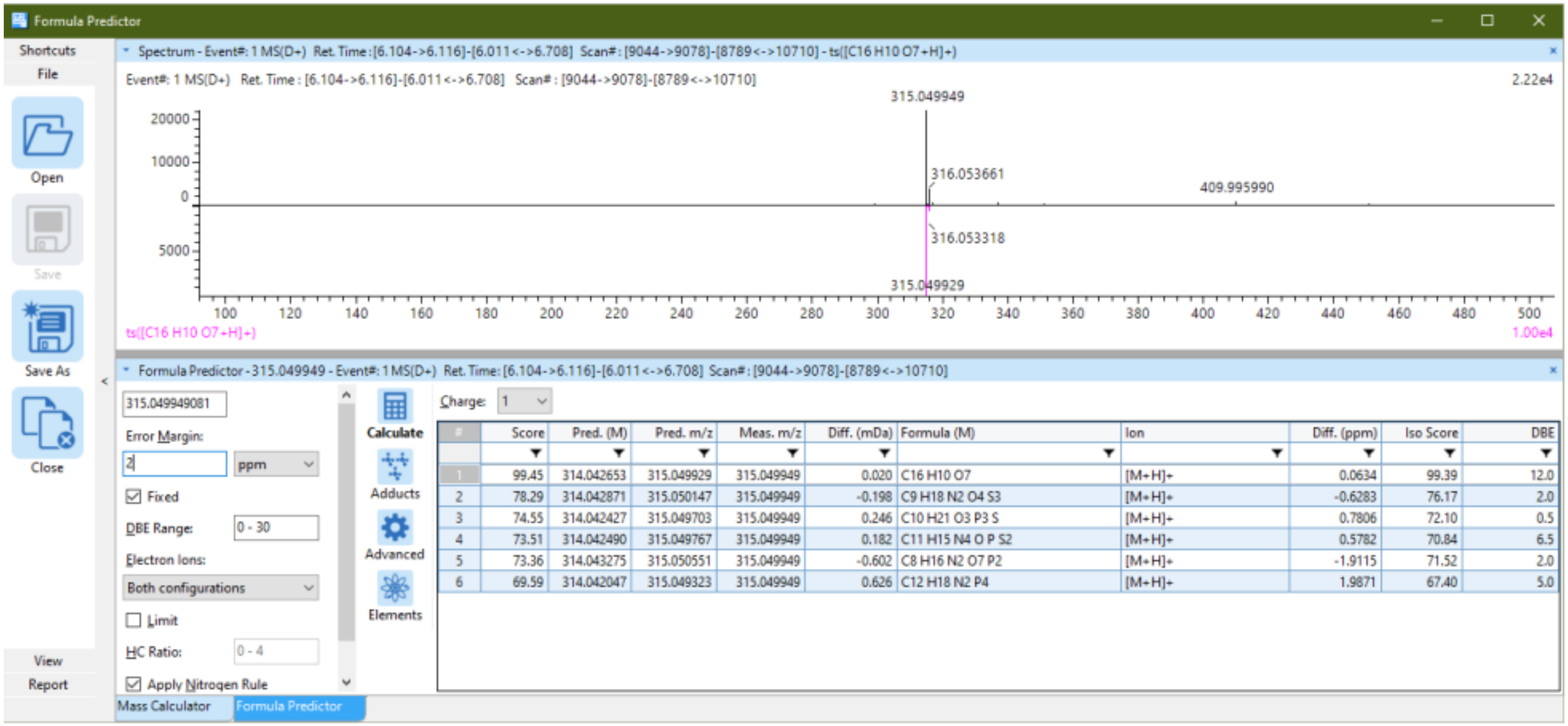

Figure S10. Chemical formula prediction of **Metabolite C**. Chemical formula was accurately predicted (< 1.0 ppm) based on the measured mass of **Metabolite C** (*m/z* 315.0499 $[M+H]^+$).

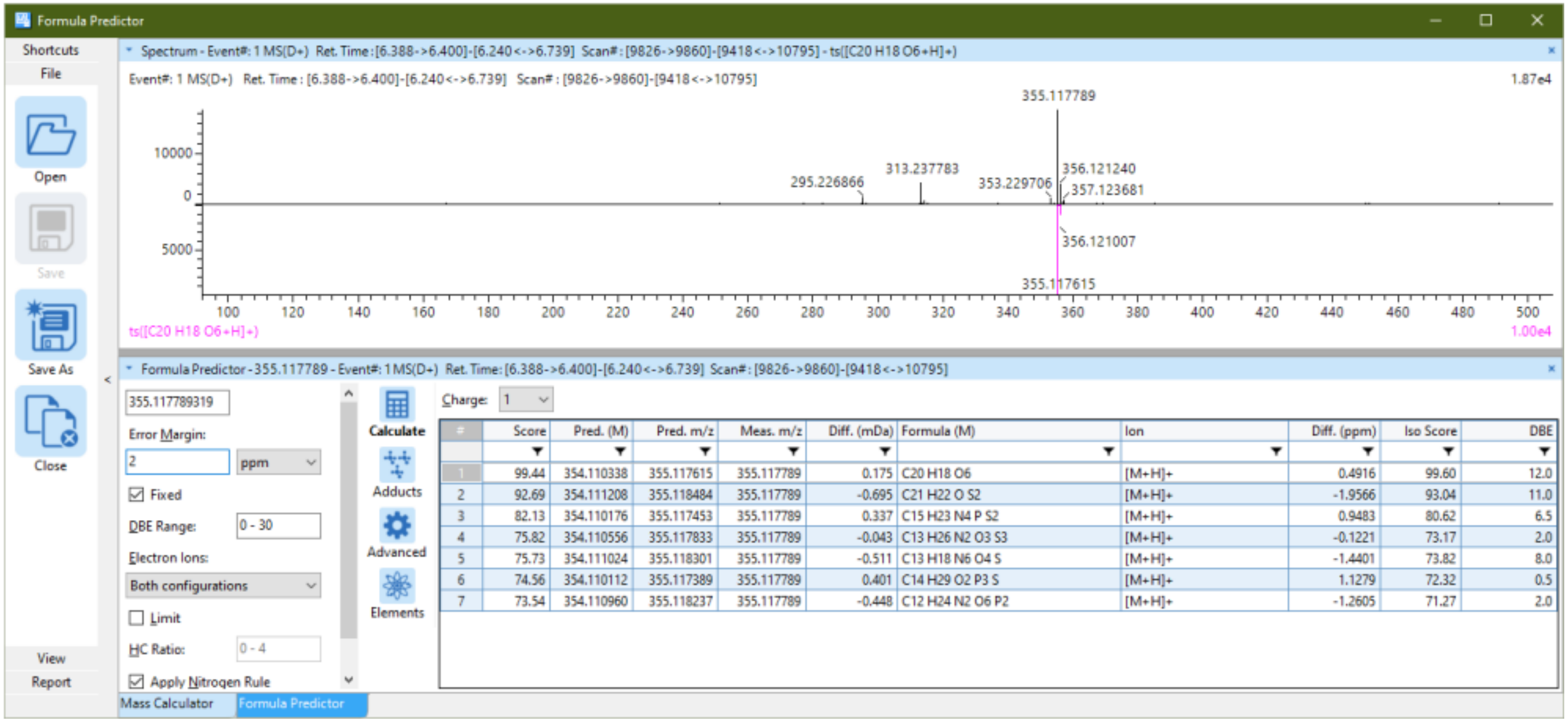

Figure S11. Chemical formula prediction of **Metabolite D**. Chemical formula was accurately predicted (< 1.0 ppm) based on the measured mass of **Metabolite D** (*m/z* 355.1178 $[M+H]^+$).

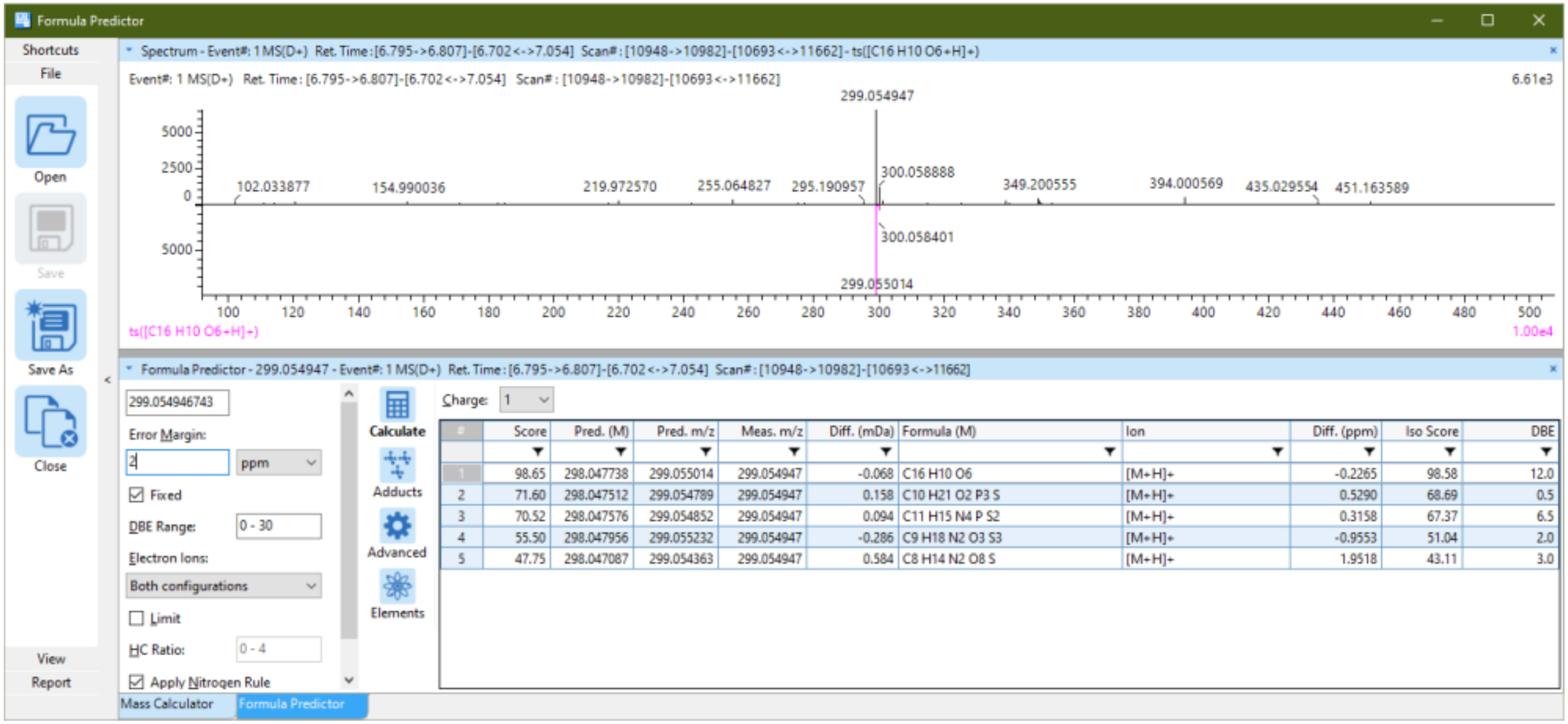

Figure S12. Chemical formula prediction of **Metabolite E**. Chemical formula was accurately predicted (< 1.0 ppm) based on the measured mass of **Metabolite E** (*m*/*z* 299.0549 [M+H]+).

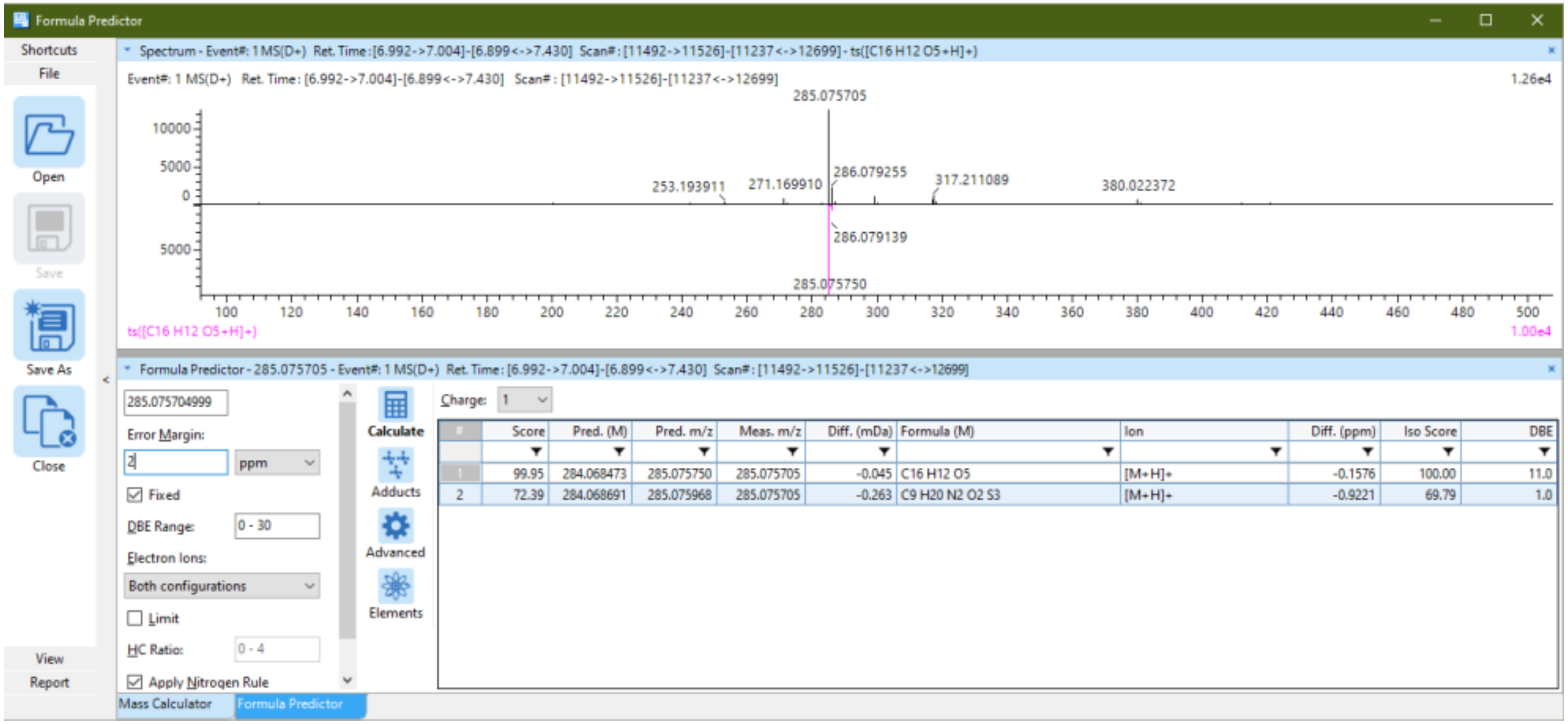

Figure S13. Chemical formula prediction of **Metabolite F**. Chemical formula was accurately predicted (< 1.0 ppm) based on the measured mass of **Metabolite F** (*m/z* 285.0757 $[M+H]^+$).

A

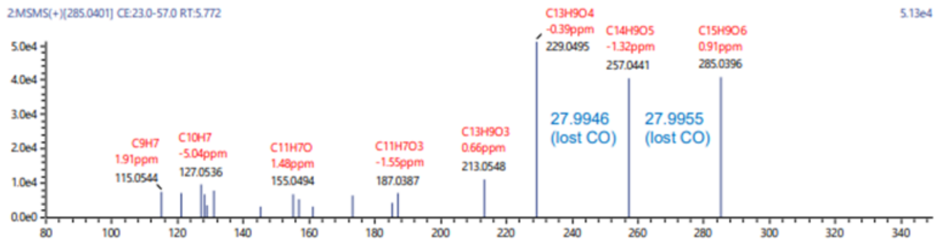

#	Name	Formula	Mass	Assign Score (%)	Structure
1	Rhein	C15H8O6	284.03207	71.1	S1
2	Lupinalbin A	C15H8O6	284.03207	71.1	S2
5	aureol	C15H8O6	284.03207	71.1	S3
10	9-Oxo-9H-xanthene-1,7-dicarboxylic acid	C15H8O6	284.03207	71.1	S4

#1, Rhein

#	m/z	Intensity	Formulae (M)	Charge	+/- ppm	Structures
21	285.0396	40959	C15H9O6	----	0.9	----
20	257.0441	40347	C14H9O5	[+H]+	-1.3	4
19	229.0495	51279	C13H9O4	[+H]+	-0.4	3
18	213.0548	11247	C13H9O3	[+H]+	0.6	2
17	187.0387	7138	C11H7O3••	[+H]+	-1.6	1
16	187.0387	7138	C11H7O3	[+H]+	-1.6	1
14	173.0595	6562	C11H9O2	[+H]+	-1.5	1
11	155.0494	6965	C11H7O••	[+H]+	1.5	1
10	155.0494	6965	C11H7O	+	1.5	1
9	145.0275	3376	C9H5O2••	[+H]+	-6.5	2
8	145.0275	3376	C9H5O2	[+H]+	-6.5	1
7	131.0484	7853	C9H7O••	[+H]+	-5.4	1
6	131.0484	7853	C9H7O	+	-5.4	1
2	121.0282	7053	C7H5O2	[+H]+	-1.8	1
1	115.0545	7571	C9H7	----	1.9	----

B

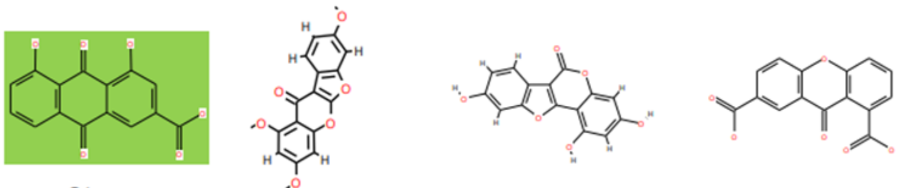

S1 S2 S3 S4

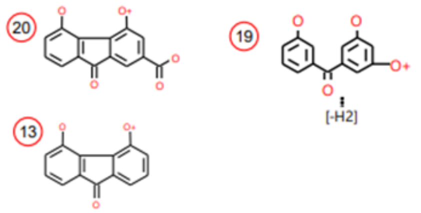

13

Figure S14. Dereplication of **Metabolite A** using High-Resolution Mass Spectrometry (HRMS) data and ChemSpider database. (**A**) MS/MS spectra of **Metabolite A**.; (**B**) Assigned identity of **Metabolite A** dereplicated as rhein using ChemSpider database.

A

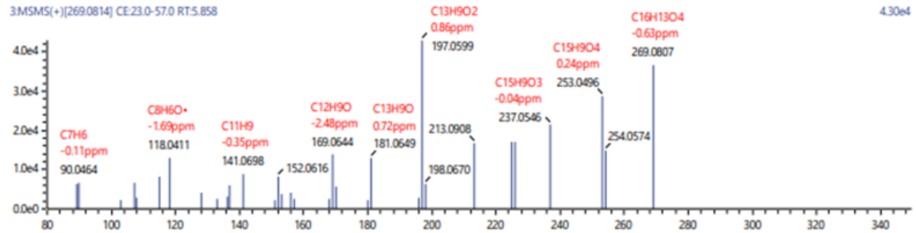

#	Name	Formula	Mass	Assign Score (%)	Structure
5	isoformononetin	C16H12O4	268.07355	100	S1
4	dalbergin	C16H12O4	268.07355	100	S2
2	Isoxepac	C16H12O4	268.07355	100	S3
1	IN00295	C16H12O4	268.07355	100	S4
10	5-Hydroxy-4'-methoxyisoflavone	C16H12O4	268.07355	100	S5

#5, isoformononetin

#	m/z	Intensity	Formulae (M)	Charge	+/- ppm	Structures
14	269.0807	36774	C16H13O4	----	-0.6	----
13	254.0574	14850	C15H10O4•	[+H]+	0.3	1
12	253.0496	28609	C15H9O4	[+H]+	0.2	1
11	237.0546	21553	C15H9O3	+	0	2
10	226.0626	16956	C14H10O3•	[+H]+ / +	0.8	4
9	225.0542	17038	C14H9O3	[+H]+ / +	-1.8	4
8	213.0908	16894	C14H13O2	----	-0.9	----
7	197.0599	42988	C13H9O2	+	0.9	2
6	181.0649	12821	C13H9O	[+H]+	0.7	1
5	169.0644	13865	C12H9O	+	-2.5	1
4	152.0616	8315	C12H8•	+	-2.8	1
3	141.0698	8738	C11H9	----	-0.3	----
2	118.0411	12909	C8H6O•	[+H]+ / +	-1.7	2
1	115.0541	8112	C9H7	----	-1	----

B

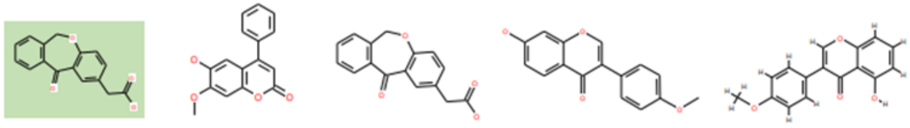

S1 S2 S3 S4 S5

Figure S15. Dereplication of **Metabolite B** using High-Resolution Mass Spectrometry (HRMS) data and ChemSpider database. (**A**) MS/MS spectra of **Metabolite B**.; (**B**) Assigned identity of **Metabolite B** dereplicated as isoformononetin using ChemSpider database.

A

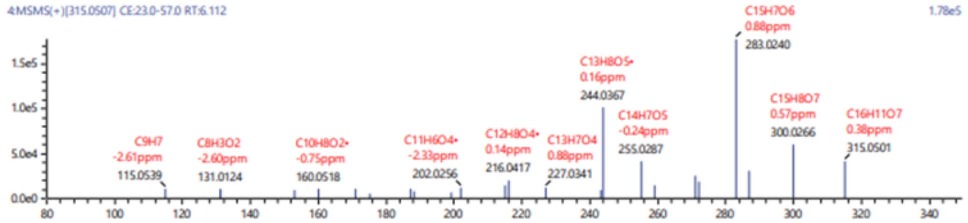

#	Name	Formula	Mass	Assign Score (%)	Structure
10	5,8-Dihydroxy-2-(2,3,4-trihydroxyphenyl)-1,4-naphthoquinone	C16H10O7	314.04266	100	S1
7	laccaic acid D	C16H10O7	314.04266	100	S2
6	3,4-Bis(3,4-dihydroxyphenyl)-2,5-furandione	C16H10O7	314.04266	100	S3
2	rhodolamprometrin	C16H10O7	314.04266	100	S4
9	terephthalic anhydride	C16H10O7	314.04266	100	

#7, laccaic acid D

#	m/z	Intensity	Formulae (M)	Charge	+/- ppm	Structures
24	315.0501	41150	C16H11O7	----	0.4	----
23	300.0266	59497	C15H8O7•	[+H]+	0.6	1
22	287.0552	30088	C15H11O6	[+H]+	0.5	4
21	283.024	177541	C15H7O6	+	0.9	2
20	272.0318	19082	C14H8O6•	[+H]+	1.1	11
19	271.0233	25106	C14H7O6	[+H]+	-1.6	4
18	259.0604	15041	C14H11O5	[+H]+	1.2	1
17	255.0287	41929	C14H7O5	[+H]+	-0.2	1
16	244.0367	101776	C13H8O5•	[+H]+	0.2	13
15	243.0291	8779	C13H7O5	[+H]+	1.1	1
14	227.0341	11550	C13H7O4	[+H]+	0.9	1
13	216.0417	20609	C12H8O4•	[+H]+	0.1	12
12	215.0337	14688	C12H7O4	[+H]+ / +	-1	13
11	202.0256	11921	C11H6O4•	[+H]+	-2.3	5
10	199.0395	6838	C12H7O3	+	2.8	2
9	188.0471	7612	C11H8O3•	[+H]+	1.4	5
8	187.0387	10341	C11H7O3	[+H]+ / +	-1.3	5
7	175.0383	5491	C10H7O3	[+H]+	-3.8	1
6	171.0438	10162	C11H7O2	+	-1.6	6
5	160.0518	10282	C10H8O2•	[+H]+	-0.8	2
4	153.0179	8838	C7H5O4	[+H]+	-2.1	1
3	131.0491	8847	C9H7O	+	-0.3	1
2	131.0124	10768	C8H3O2	+	-2.6	3
1	115.0539	10506	C9H7	----	-2.6	----

B

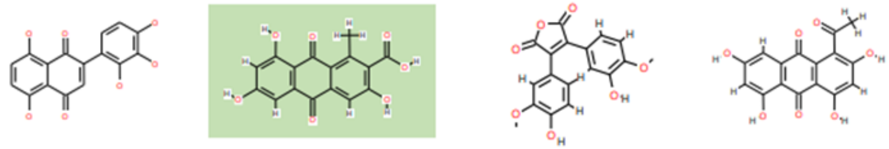

S1 S2 S3 S4

Figure S16. Dereplication of **Metabolite C** using High-Resolution Mass Spectrometry (HRMS) data and ChemSpider database. (**A**) MS/MS spectra of **Metabolite C**.; (**B**) Assigned identity of **Metabolite C** dereplicated as laccaic acid D using ChemSpider database.

A

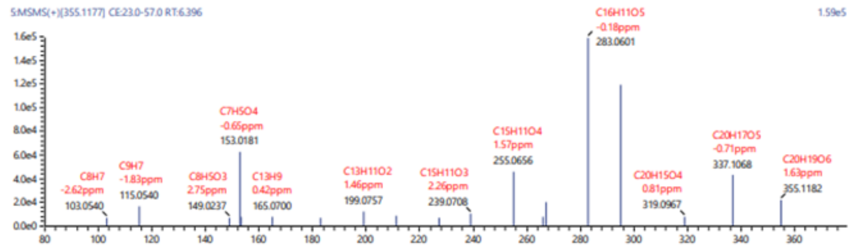

#	Name	Formula	Mass	Assign Score (%)	Report
5	crotafuran E	C20H18O6	354.11035	100	S1
4	Ephedroidin	C20H18O6	354.11035	100	S2
10	2,6-Anhydro-1,4-di-O-benzoyl-5-deoxy-D-arabino-hex-5-enitol	C20H18O6	354.11035	100	
9	Lico-iso-flavanone	C20H18O6	354.11035	100	
8	Hydroxytuberosin	C20H18O6	354.11035	100	
7	Sesamin	C20H18O6	354.11035	100	

#5, crotafuran E						
#	m/z	Intensity	Formulae (M)	Charge	+/- ppm	Structures
19	355.1182	22441	C20H19O6	----	1.6	----
18	337.1068	42929	C20H17O5	+	-0.7	6
17	319.0968	7774	C20H15O4	+	0.8	12
16	295.06	119024	C17H11O5	[+H]+	-0.4	1
15	283.0601	159050	C16H11O5	[+H]+ / +	-0.2	5
14	267.0654	20176	C16H11O4	[+H]+ / +	0.6	14
13	266.0573	7518	C16H10O4•	[+H]+ / +	-0.4	2
12	255.0656	46088	C15H11O4	[+H]+	1.6	1
11	239.0708	10968	C15H11O3	+	2.2	1
10	227.0701	7118	C14H11O3	[+H]+	-0.7	1
9	211.075	8682	C14H11O2	[+H]+ / +	-2	4
8	199.0757	12338	C13H11O2	[+H]+ / +	1.5	7
7	183.0804	7103	C13H11O	+	-0.1	1
6	165.07	7947	C13H9	----	0.5	----
5	153.0699	7732	C12H9	----	-0.1	----
4	153.0181	62662	C7H5O4	[+H]+	-0.7	1
3	149.0237	7359	C8H5O3	[+H]+	2.8	3
2	115.054	17182	C9H7	----	-1.9	----
1	103.054	7206	C8H7	----	-2.6	----

B

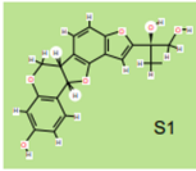
S1

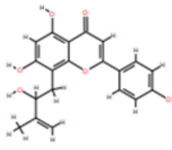
S2

22

Figure S17. Dereplication of **Metabolite D** using High-Resolution Mass Spectrometry (HRMS) data and ChemSpider database. (**A**) MS/MS spectra of **Metabolite D**.; (**B**) Assigned identity of **Metabolite D** dereplicated as crotafuran E using ChemSpider database.

A

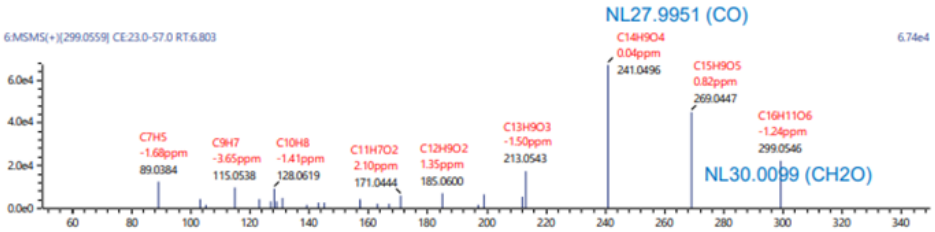

#	Name	Formula	Mass	Assign Score (%)	Report
13	8,9-Dihydroxy-10-methoxy-6H-[1]benzofuro[3,2-c]chromen-6-one	C16H10O6	298.04773	100	S1
3	Ayamenin A	C16H10O6	298.04773	100	S2
1	4,5'-Bi-1,3-benzodioxole-5,6'-dicarbaldehyde	C16H10O6	298.04773	100	S3
19	5-hydroxypseudobaptigenin	C16H10O6	298.04773	100	
18	1,2-Bis(1,3-benzodioxol-5-yl)-1,2-ethanedione	C16H10O6	298.04773	100	

#3, Ayamenin A						
#	m/z	Intensity	Formulae (M)	Charge	+/- ppm	Structures
16	299.0546	22544	C16H11O6	----	-1.2	----
15	269.0447	44912	C15H9O5	[+H]+	0.8	5
14	241.0496	67376	C14H9O4	[+H]+ / +	0.1	11
13	213.0543	17659	C13H9O3	+	-1.5	2
12	212.0471	5332	C13H8O3•	[+H]+	1.4	4
11	199.0396	6424	C12H7O3	[+H]+	3	1
10	185.06	7288	C12H9O2	----	1.3	----
9	171.0444	5932	C11H7O2	[+H]+ / +	2.1	2
8	157.0644	4368	C11H9O	----	-2.7	----
7	131.0488	4747	C9H7O	----	-3	----
6	128.0619	8971	C10H8	----	-1.4	----
5	127.0532	3376	----	----	----	----
4	123.0073	4235	C6H3O3	+	-3.4	4
3	115.0538	9547	C9H7	----	-3.7	----
2	103.054	4194	C8H7	----	-2.6	----
1	89.03843	12682	C7H5	----	-1.7	----

B

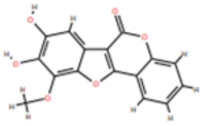

S1

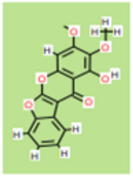

S2

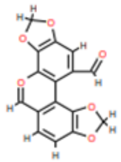

S3

24

Figure S18. Dereplication of **Metabolite E** using High-Resolution Mass Spectrometry (HRMS) data and ChemSpider database. (**A**) MS/MS spectra of **Metabolite E**.; (**B**) Assigned identity of **Metabolite E** dereplicated as ayamenin A using ChemSpider database.

A

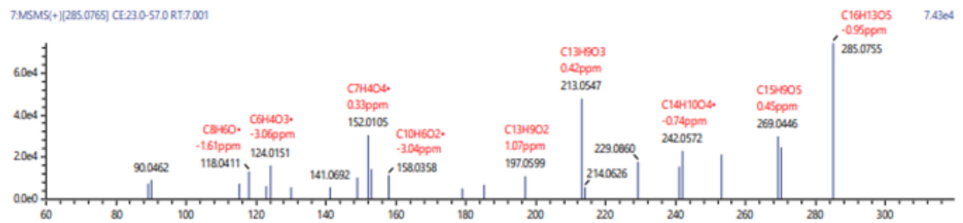

#	Name	Formula	Mass	Assign Score (%)	Structure
1	olmelin	C16H12O5	284.06848	79.2	S1
2	Glycitein	C16H12O5	284.06848	91.7	S2
3	Parietin	C16H12O5	284.06848	75	S3
4	Wogonin	C16H12O5	284.06848	83.3	S4
5	Acacetin	C16H12O5	284.06848	87.5	S5
6	Genkwanin	C16H12O5	284.06848	91.7	S6
7	oroxylin A	C16H12O5	284.06848	83.3	S7
8	Prunetin	C16H12O5	284.06848	91.7	S8
9	Calycosin	C16H12O5	284.06848	91.7	S9
13	negletein	C16H12O5	284.06848	87.5	S10

#1, olmelin (Biochanin A)

#	m/z	Intensity	Formulae (M)	Charge	+/- ppm	Structures
15	285.0755	74303	C16H13O5	----	-0.9	----
14	270.0522	24550	C15H10O5•	[+H]+	-0.1	1
13	269.0446	29885	C15H9O5	[+H]+	0.5	1
12	253.0495	21482	C15H9O4	[+H]+ / +	-0.2	2
11	242.0572	23153	C14H10O4•	[+H]+	-0.7	4
10	241.0495	15726	C14H9O4	[+H]+	-0.2	4
9	229.086	17803	C14H13O3	----	0.5	----
8	213.0547	47574	C13H9O3	[+H]+	0.4	3
7	197.0599	11021	C13H9O2	[+H]+ / +	1.1	4
6	158.0358	11624	C10H6O2•	[+H]+	-3	2
5	153.0181	14324	C7H5O4	[+H]+	-0.9	2
4	152.0105	30756	C7H4O4•	[+H]+	0.3	4
3	149.0231	10218	C8H5O3	+	-1.8	2
2	124.0151	15926	C6H4O3•	[+H]+ / +	-3.1	7
1	118.0411	13329	C8H6O	----	-1.6	----

B

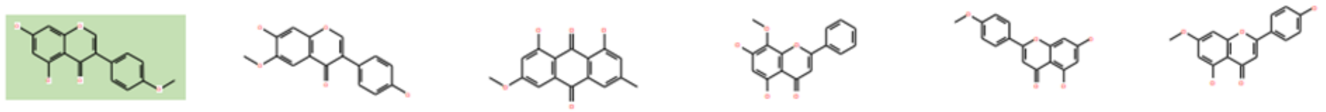

S1 S2 S3 S4 S5 S6

Figure S19. Dereplication of **Metabolite F** using High-Resolution Mass Spectrometry (HRMS) data and ChemSpider database. (**A**) MS/MS spectra of **Metabolite F**.; (**B**) Assigned identity of **Metabolite F** dereplicated as olmelin or biochanin A using ChemSpider database.

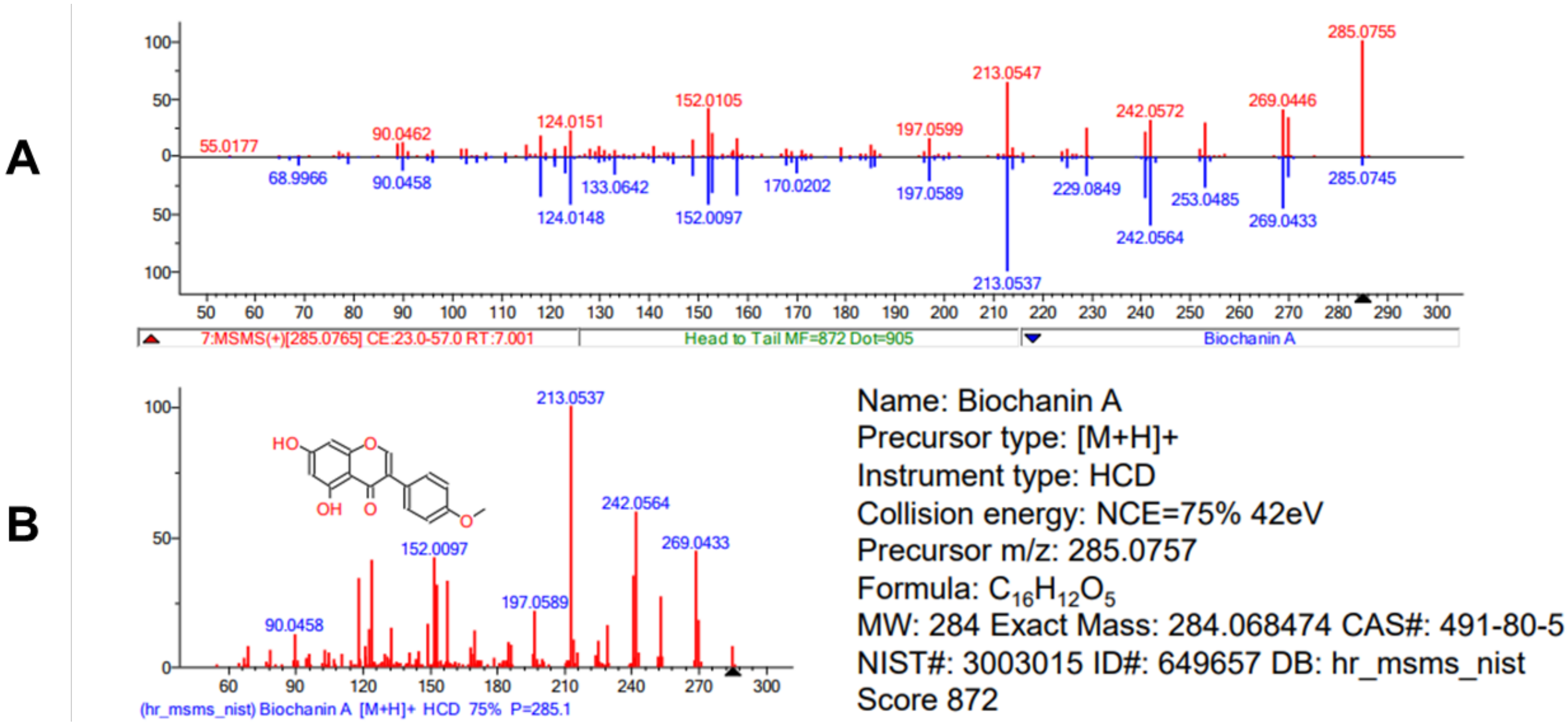

Figure S20. Dereplication of **Metabolite F** using High-Resolution Mass Spectrometry (HRMS) data and National Institute of Standards and Technology (NIST) Library. (**A**) MS/MS spectra of **Metabolite D**.; (**B**) Assigned identity of **Metabolite F** dereplicated as biochanin A using NIST Library.

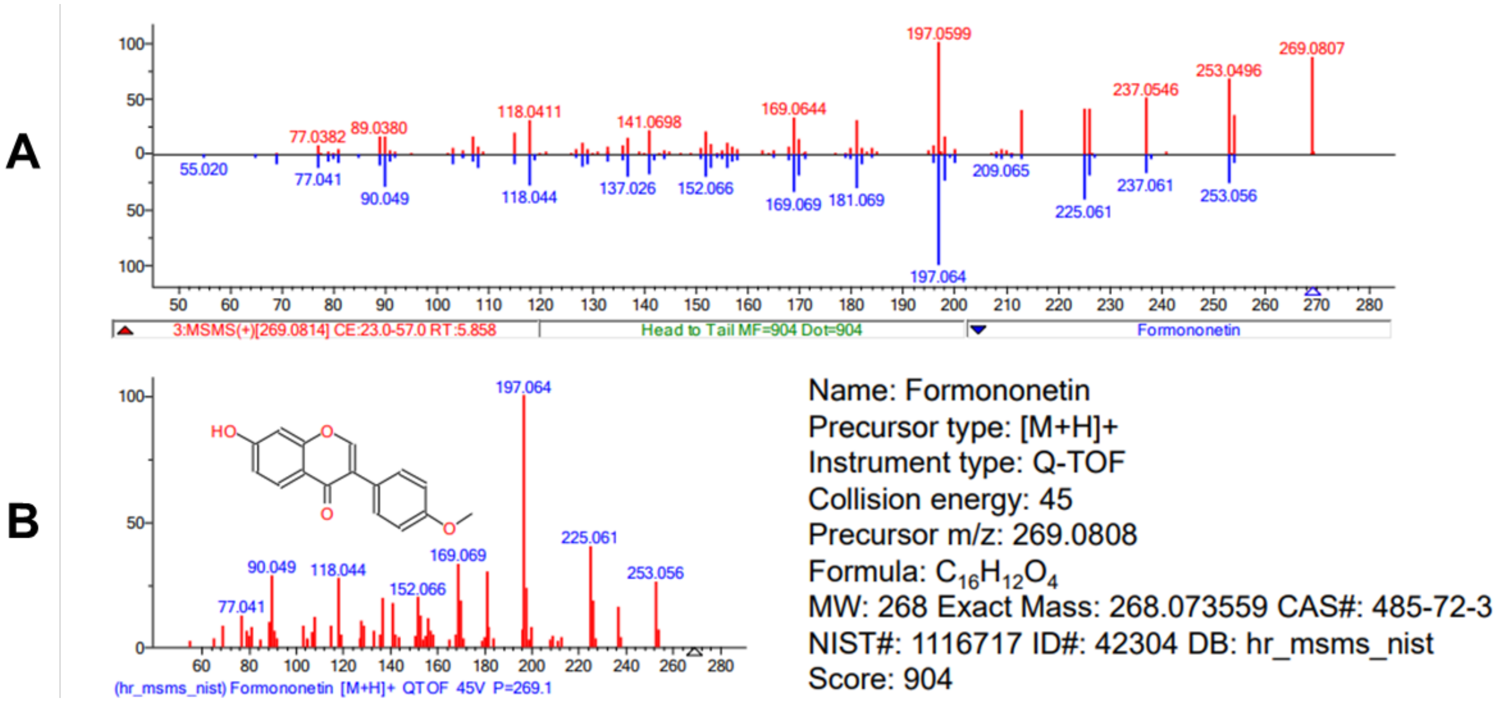

Figure S21. Dereplication of **Metabolite B** using High-Resolution Mass Spectrometry (HRMS) data and National Institute of Standards and Technology (NIST) Library. (**A**) MS/MS spectra of **Metabolite B**.; (**B**) Assigned identity of **Metabolite B** dereplicated as formononetin using NIST Library.

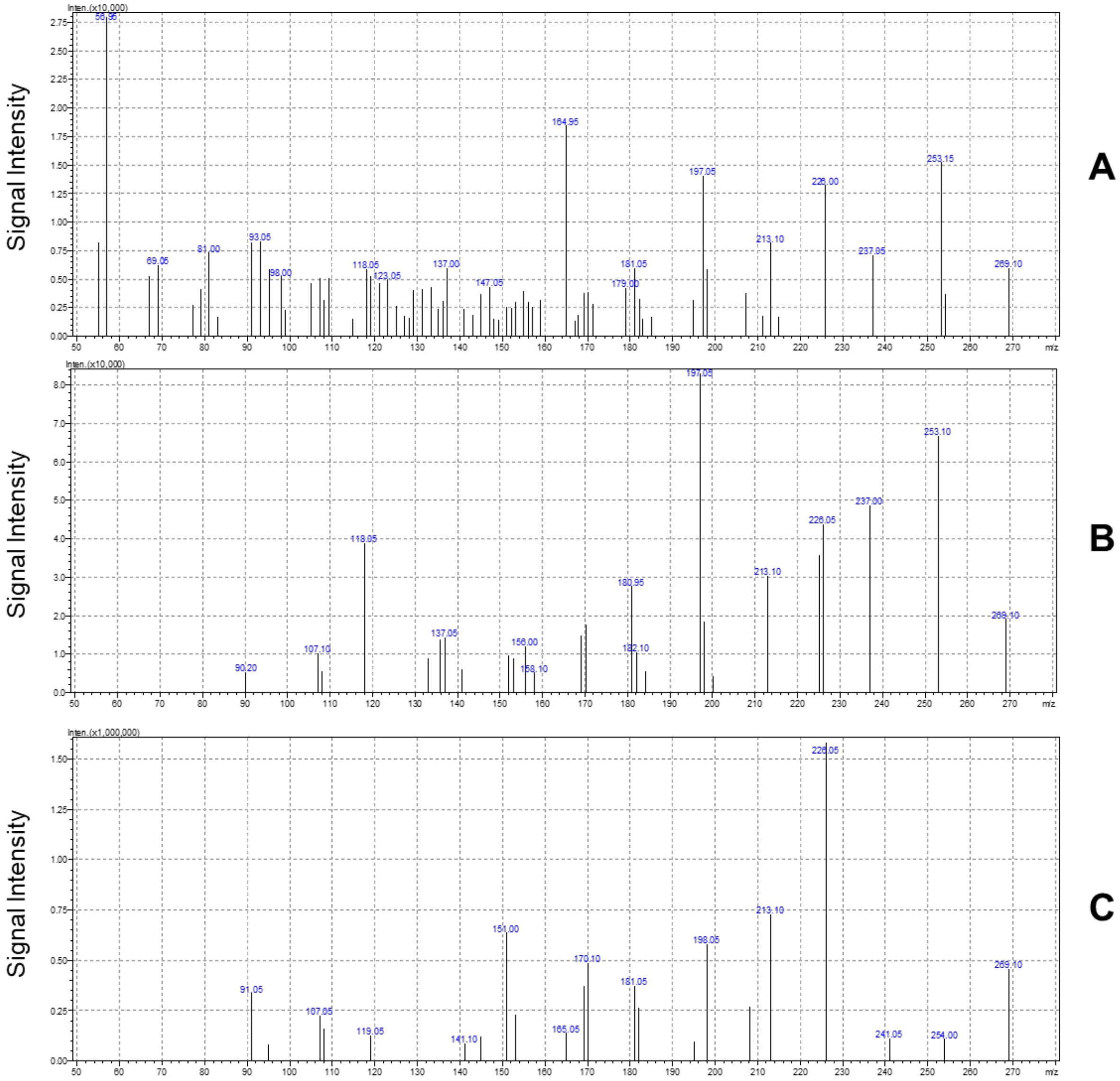

Figure S22. Comparison of MS/MS Fragmentation Pattern of Metabolite B from fraction KADAMEI3I7H2 with standards of isomers formononetin and isoformononetin. (**A**) MS/MS spectra of Metabolite B; (**B**) standard formononetin; (**C**) isoformononetin each injected at 2.0 μg (3.0 μL). Collision Energy was held constant at -32.5 V
